# The Role of Immunological Synapse in Predicting the Efficacy of Chimeric Antigen Receptor (CAR) Immunotherapy

**DOI:** 10.1186/s12964-020-00617-7

**Published:** 2020-08-25

**Authors:** Dongfang Liu, Saiaditya Badeti, Gianpietro Dotti, Jie-gen Jiang, He Wang, James Dermody, Patricia Soteropoulos, Deanna Streck, Raymond B. Birge, Chen Liu

**Affiliations:** 1 Department of Pathology, Immunology and Laboratory Medicine, Rutgers University- New Jersey Medical School, 185 South Orange Avenue, Newark, NJ 07103 USA; 2 Center for Immunity and Inflammation, New Jersey Medical School, Rutgers-The State University of New Jersey, Newark, NJ 07101 USA; 3 Department of Microbiology and Immunology and Lineberger Comprehensive Cancer Center, University of North Carolina, Chapel Hill, NC 27599 USA; 4 Institute of Genomic Medicine, New Jersey Medical School, Rutgers-The State University of New Jersey, Newark, NJ 07103 USA; 5 Department of Microbiology, Biochemistry and Molecular Genetics, New Jersey Medical School, Rutgers-The State University of New Jersey, Newark, NJ 07103 USA; 6 Department of Pathology, Yale School of Medicine, Yale University, 333 Cedar Street, New Haven, CT 06510 USA

**Keywords:** chimeric antigen receptor, immunotherapy, immunological synapse, and cancer

## Abstract

**Abstract:**

Chimeric Antigen Receptor (CAR) immunotherapy utilizes genetically-engineered immune cells that express a unique cell surface receptor that combines tumor antigen specificity with immune cell activation. In recent clinical trials, the adoptive transfer of CAR-modified immune cells (including CAR-T and CAR-NK cells) into patients has been remarkably successful in treating multiple refractory blood cancers. To improve safety and efficacy, and expand potential applicability to other cancer types, CARs with different target specificities and sequence modifications are being developed and tested by many laboratories. Despite the overall progress in CAR immunotherapy, conventional tools to design and evaluate the efficacy and safety of CAR immunotherapies can be inaccurate, time-consuming, costly, and labor-intensive. Furthermore, existing tools cannot always determine how responsive individual patients will be to a particular CAR immunotherapy. Recent work in our laboratory suggests that the quality of the immunological synapse (IS) can accurately predict CAR-modified cell efficacy (and toxicity) that can correlate with clinical outcomes. Here we review current efforts to develop a Synapse Predicts Efficacy (SPE) system for easy, rapid and cost-effective evaluation of CAR-modified immune cell immunotherapy. Ultimately, we hypothesize the conceptual basis and clinical application of SPE will serve as an important parameter in evaluating CAR immunotherapy and significantly advance precision cancer immunotherapy.

**Video abstract**

**Graphical abstract:**

Graphic abstract for manuscript CCAS-D-20-00136 by Liu, D., et al., ‘The Role of Immunological Synapse in Predicting the Efficacy of Chimeric Antigen Receptor (CAR) Immunotherapy”. The various branches of evaluating cancer immunotherapy metaphorically represented as a Rubik’s cube. The development of a novel approach to predict the effectiveness of Chimeric Antigen Receptor (CAR)-modified cells by quantifying the quality of CAR IS will introduce a new parameter to the rapidly expanding field of cancer immunotherapy. Currently, no single parameter can predict the clinical outcome or efficacy of a specific type of CAR-modified cell. IS quality will serve as a quantifiable measure to evaluate CAR products and can be used in conjunction with other conventional parameters to form a composite clinical predictor. Much like a Rubik’s cube has countless configurations, several methods and combinations of clinical metrics have arisen for evaluating the ability of a given immunotherapeutic strategy to treat cancer. The quality of IS depicting cancer immunotherapy is metaphorically expressed as a Rubik’s cube. Each face/color represents one aspect of cancer therapy. Each grid in one face indicates one factor within that aspect of cancer therapy. For example, the green color represents the tumor microenvironment, and one out of the nine grids in the green color indicates suppressor cells (suppressors in green). Changes in one factor may completely alter the entire strategy of cancer therapy. However, the quality of IS (illuminated center red grid) makes the effectiveness of CAR immunotherapy predictable. 
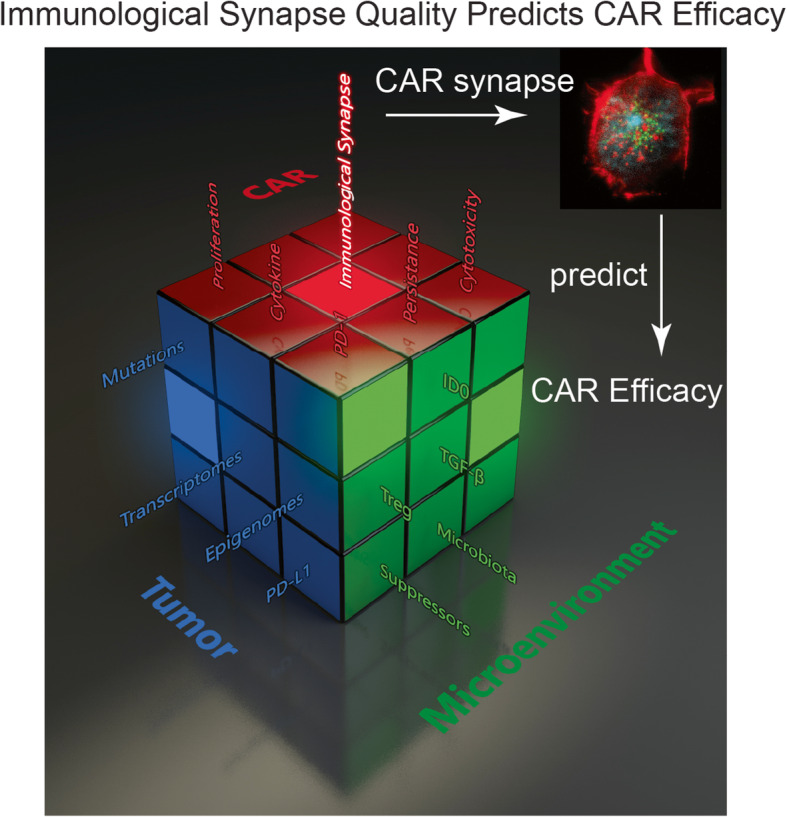

## Immunotherapy and Chimeric Antigen Receptors (CAR)

Immunotherapy, mainly defined as harnessing a patient’s own or third party’s immune cells to target tumors, has become a mainstream and powerful treatment option in cancer biology and immunotherapy [1–3]. One prominent advance, engineered Chimeric Antigen Receptor T (CAR-T) cells, was conceptually pioneered in the late 1980s by Zelig Eshhar [4], and has emerged as a frontline therapeutic modality in immuno-oncology and immunotherapy [5]. Most emblematically, CD19-CAR-T and CD19-CAR-NK, CARs with optimal co-stimulatory signaling and clinical efficacy, have provided an impetus for additional research in both cancer and infectious diseases [6, 7], showing demonstrated beneficial outcomes in patients with B cell lymphoma [8, 9] relapsed or refractory CD19-positive non-Hodgkin's lymphoma, and chronic lymphocytic leukemia [CLL]) [10], CAR-T cell immunotherapy has also shown promising results for multiple myeloma [11, 12], leukemia [9, 13–15], sarcoma [16], and neuroblastoma [17, 18]. These findings have led pharmaceutical companies and academic laboratories to extend significant investments in CAR-T or CAR-NK cell immunotherapy [19–22]. The history of CAR-T cell development [23], design [24–26], optimization [27], therapeutic resistance and challenges [2, 28], and CAR-T basic research and clinical progress have been reviewed elsewhere [29, 30] and beyond the scope of the current review.

Despite the mainstay of CAR-T biology in immune-oncology, an array of current efforts are underway to both improve biological efficacy and minimize side effects and toxicity [31, 32]. For example, while results of clinical trials employing CD19-CAR-T immunotherapy are clearly encouraging, 10-36% of these highly-selected study patients do not respond, depending on the specific CAR construct [33–35]. Furthermore, CAR immunotherapy itself is often associated with significant toxicity and relapse [36–40]. Given such risks, the high cost of immunotherapy [41], and the expanding number of CAR constructs in development, it becomes imperative to predict which CAR constructs will be most effective for a given cancer patient in a cost-effective and timely manner in the era of precision medicine.

In this review, we discuss challenges inherent in current methodologies for bringing new CAR-modified immune cells from laboratory to clinic, focusing on current strategies in basic research for evaluating CAR-modified immune cell efficacy. Subsequently, we propose novel, better time and better cost-efficient methodologies, based on the conceptual idea that the immunological synapse (IS) quality, defined as a single-cell level of communication between an immune cell and a tumor cell, can be a predictive biomarker for CAR efficacy and toxicity in laboratory studies and in clinical applications [42–46]. As such, the goal of this review is to put forward the idea that (i) the quality of the CAR-T or CAR-NK cell IS can be an accurate predictor of the antitumor activity of CAR-modified immune cells and (ii) IS technology can form the basis of the development of fast, easy, and inexpensive tools to predict CAR-T-cell effectiveness or toxicity in cancer patients. IS quality evaluation also promises to establish a reliable standard for side-by-side comparison of CAR products from different commercial sources prior to injection into cancer patients. Below, we develop the rationale and current status of IS evaluation in CAR-T biology. In the following sections, we discuss current conventional methods to predict efficacies and potential utilities of CAR-modified immune cells, as well challenges that exist and needed to be addressed to reduce toxicities in patients by evaluating the IS quality.

## Conventional Methods to Predict Efficacy of CAR Cells

Traditionally, following design and generation of CAR-T cells (both academic research laboratories and/or industrial scale-ups), ranking the efficacy of CAR-modified immune cell products has been hampered by a number of time-consuming, costly, and labor-intensive conventional tools used to evaluate efficacy *in vivo* (Table 1).

**Table 1:** Comparison of currently available methods for evaluating CAR efficacy in research lab and in clinic

Method Types	Currently available Methods	Assay readouts	Pros	Cons
In vitro assays	**Immunophenotyping assay**	ratio of CD4 and CD8	Easy and quick	Cannot be used a predictive biomarker due to variations from each individual.
				Cannot reflect the phenotyping in vivo
	**Proliferation and Cytokine secretion assay**	IL-2 and IFN-gamma productions	Easy and quick	Cannot reflect the proliferation and cytokine productions in vivo
	**Cytotoxicity by standard** ^**51**^**Cr-release assay**	4-hour killing of tumor cells	Easy and quick	Short -term in vitro activation
				No interaction with host real tumor cells
	**Long-term killing assay**	Number of CAR-T cells	Reflects the CAR-T expansion and tumor killing in 1- or 2-week in vitro assay	Time-consuming
		Number of artificial tumor cells		Artificial modified tumor cell lines
				Slow and variable
				Technically complex
	**Available** ***in vitro*** **Strategies for clinical use CAR-T cells**	Vector copy number (VCN) and CAR expression	Technically convenient	Cannot be used as predictive biomarker due to variations from each individual.
In vivo assays	**NSG mouse model**	Tumor size and tumor growth	Predicts persistence of CAR cells	No host immune system
		Mouse survival	Predicts CAR-T killing capability	No tumor microenvironment (TME)
		Body weight	Easy to use	Expensive
				Labor-intensive
	**Syngeneic transplantable model**	Tumor size	Intact host immune system	Slow and complex
		Mouse survival	Some TME development	Model is variable
		Body weight		Expensive
		Biodistribution of CAR cells		Labor-intensive

As such, while current approaches can provide useful information concerning the validity of the functional CAR, these methodologies often fail to predict the efficacy of CAR-modified immune cells *in vivo*. The lack of a universal standard drastically complicates the evaluation of product utility and functional outcomes. Clearly, efforts to normalize CAR-T testing in a unified and consensus assay, particularly in the current era of precision medicine, would be a welcome advance to the field.

As shown in Table 1, several *in vitro* approaches are currently employed to assess CAR efficacy that include; (i) immunophenotyping, (ii) proliferation and cytokine release, (iii) chromium release (direct cytotoxicity), (iv) long-term killing assays and (v) interferon gamma (IFN-γ) production. While each has some intrinsic merit with respect to potential prediction of functional activity, all are *in vitro* assays, and have to be extrapolated for *in vivo* utility. Moreover, our published data as well as those of other groups show that conventional cytokine-based assays (e.g., IL-2 and IL-6), CD4/CD8, and Cr^51^ release assays do not predict CAR-T *in vivo* efficacy [47, 48] potentially limiting the utility of these assays to *in vivo* performance. We compare the currently available parameters in the Table 2.

**Table 2:** Summary of currently available parameters for predicting the efficacy of CAR-modified immune cells

	Parameter	Prediction	Disease	References
Pre-expansion of T cells	Percentage of pre-expansion CD8 T_N_ (naive), CD8 T_CM_ (central memory), T_EM_ (effector memory), T_EFF_ (effector)	No	CLL	[49]
	Percentage of pre-expansion CD8 T_SCM_ (stem cell memory)	Modestly significant	CLL	[49]
	Percentage of pre-expansion CD45RO^-^ CD27^+^ CD8^+^ T cells	Yes	CLL	[49]
CAR cells	Infused CAR cell dose	No	CLL	[49]
	CD27^+^ PD-1^-^CD8^+^ CAR-T cells	Yes	CLL	[49]
	Upregulated pSTAT3 in response to IL-6 from CD27^+^ PD-1^-^ CD8^+^ CAR-T cells with IL-6 receptor-β chain	Yes	CLL	[49]
	CD4/CD8 ratio	No	CLL	[49]
	CAR transduction efficiency	No	CLL	[47, 49]
	Transgene level in blood	No	CLL	[49]
	Telomere length of CAR	No	CLL	[49]
	CAR RNA-sequence	Yes	CLL	[49]
Cytokine	Serum IL-6 level	Debated	CLL	[48, 49]
	Serum IL-15 level	Yes	DLBCL	[48]
	Serum IL-10 level	Yes	DLBCL	[48]
	4-h Cr^51^ release	No	Not Applicable (NA)	[47]
	IFN-gamma (IFN-γ)	No	Not Applicable (NA)	[47]
	TNF-gamma	No	Not Applicable (NA)	[47]
	IL-2	No	Not Applicable (NA)	[47]
Others	Cytokine used in culture (IL-2 vs IL-7+IL-15)	No	Kappa (K^+^) NHL	[34]
	Quality of immunological synapse	Yes	Not Applicable (NA)	[47]
	In Vivo Animal model	Yes	CLL	[47, 49]

The currently available strategies to evaluate the effectiveness of CAR-T cells include conventional *in vitro* methods, such as immunophenotyping assay, proliferation and cytokine secretion assays, cytotoxicity assay, and long-term killing assays, as well as *in vitro* strategies for clinical use CAR-T cells (including vector copy number testing), as detailed below:

### Immunophenotyping assay

The growth kinetics and immunophenotye of CAR-T cells are typically measured for a minimum of 2-3 weeks. Different research laboratories use different time periods for evaluating growth kinetics, different components of CAR-T cells (e.g., ratio of CD4 and CD8 CAR positive T cells) and immunophenotye of CAR-T cells. This method ensures that CAR-modified T cells retain phenotypic and functional characteristics similar to those of non-transduced cytotoxic T lymphocytes (CTLs) [50].

### Proliferation and cytokine secretion assay

After examining the immunophenotye and composition of CAR-T cells, researchers typically examine whether transduction with CAR affects T cell proliferation and cytokine production [50–53].

### Cytotoxicity by standard ^51^Cr-release assay

A standard 4-hour ^51^Cr-release assay is the most common method to evaluate the cytotoxicity of CAR-T cells *in vitro* [50]. Some laboratories also use a luciferase killing assay or other non-radiative assays (e.g., CD107a assays) to evaluate cytotoxicity of CAR-T cells. However, the ^51^Cr-release assay is the most reliable method so far.

### Long-term killing assay

Previous studies have shown that the antitumor activity of CAR-T cells depends on long-term CAR-T cell activation, persistence, and proliferation [54]. To evaluate the antitumor activity of CAR-T cells, a long-term, *in vitro* killing assay are commonly used [50, 55]. There are also other less common approaches, such as proteomics [56] and sequence tools [49] as surrogates for killing efficacy.

### IFN-γ production and *in vitro* strategies for clinical use CAR-T cells

In the FDA briefing document “Oncologic Drugs Advisory Committee Meeting for Tisagenlecleucel (Kymriah, https://www.fda.gov/media/106081/download),” IFN-γ production in response to tumor antigen-bearing cells, transduction efficiency (Vector copy number [VCN] and CAR expression), and T cell subsets are suggested. However, the results are not recommended as criteria for CAR-T efficacy prediction.

In addition to the aforementioned *in vitro* cell based assays, currently available *in vivo* methods for evaluating the efficacy of CAR-modified immune cells include xenogeneic transplantable mouse models, which mainly employ immune-compromised NOD/SCID/gamma chain^-/-^ (NSG) mice that lack T cells, B cells, and NK cells. These NSG xenograft mouse models are relatively quick and easy, and can monitor homing, trafficking, persistence, and anti-tumor activity of CAR-modified immune cells *in vivo* in live animals using *in vivo* imaging systems [50, 57–60]. While NSG models are the most commonly used in the field of CAR immunotherapy, there are obvious shortcomings. Notably, NSG mouse models cannot evaluate the effects of tumor microenvironment (TME) and toxicity of CAR-modified immune cells including induction of cytokine storm. Traditionally, these *in vivo* NSG animal models have mainly focused on blood cancers. For solid tumors, it is even more challenging to evaluate the efficacy of CAR-modified cells in clinically relevant mouse models, which do not fully recapitulate humans [61] and such technology cannot be used in the clinical test laboratory for evaluating the efficacy of CAR-modified cells for each individual patient. A summary and comparison of strategies and challenges for both preclinical *in vitro* studies and evaluating the efficacy of CAR-modified immune cells *in vivo* is summarized in Table 1.

Collectively, while each of the aforementioned *in vitro* and *in vivo* methods to introduce CAR modified cells into patients have merit, the heterogeneity in methods (including inconsistency between laboratories) and their time-consuming, expensive, labor-intensive approaches can be problematic for transferring CAR activity to *in vivo* efficacy for each patient. Other potential problems with current *in vitro* CAR testing include the fact that there are no consensus supportive tools, services, or commercial products to evaluate CAR efficacies (most of the assays in Table 1 and Table 2 are carried out in individual laboratories), as well as there is considerable variability in the overall yields and scalability of the final products. Finally, there is considerable intrinsic complexity and variability of CAR-T products, such as unique linkers, different co-stimulating molecules, different genetically modified vectors for generating CAR-T cells, metabolic state of CAR-T cells, and expansion techniques. The complexity of CAR-modified cell products further make the reproducibility of CAR-T products challenging. Clearly, current strategies cannot be used to quickly screen the manufacturing process and CAR constructs from the CRO (Contract Research Organization)/CMO (Contract Manufacturing Organization) industry due to the lack of high-throughput capability, accuracy, and reproducibility.

The above considerations are also highlighted from the fact even in best case scenario’s that employ CAR-Ts, such as clinical trials for Acute lymphoblastic leukemia (ALL), Chronic lymphocytic leukemia (CLL), Diffuse large B-cell lymphoma (DLBCL), and non-Hodgkin lymphoma (NHL), still between 10-36% of even these highly selected study patients do not respond to CAR-T therapy.

CD19-CAR-modified T-cells (CAR-T) have been remarkably successful at treating ALL [62]. With lymphodepletion, institutional clinical trial success objective response rates (calculated as combined complete plus partial response rates) range from 64-82% in diffuse large B-cell lymphoma [33] (DLBCL) and from 85-90% in relapsed and refractory ALL on the basis of published data in USA [13–15], depending on the CAR construct tested. With such a high clinical response rate, no study has been reported to predict the clinical response for ALL treatment using CD19-CAR-T cells.

## IS Quality as a Biomarker for CAR-T Cell-Mediated Efficacy

Due to the lack of uniformly available strategies for predicting CAR efficacy in the clinic, we first proposed to utilize a conceptually novel approach that assessed quality of the IS formed by CAR-modified immune cells in order to predict efficacy and toxicity of CAR cells in basic research and clinical applications [47, 63]. As such, we posit that accurately assessing IS quality will be a powerful utility for precisely determining the best CAR therapy for a given patient, and that *in vitro* CAR-T IS quality will serve as a proxy for CAR-T effectiveness via a Synapse Predicts Efficacy (SPE) system for rapid (< 24 hours) evaluation of CAR-modified immune cells (including CAR-T and CAR-NK cells, as well as other types of CAR-modified immune cells). Importantly, this minimally instrumented system can be portable and operable by users with minimal expertise.

**Fig. 1:**
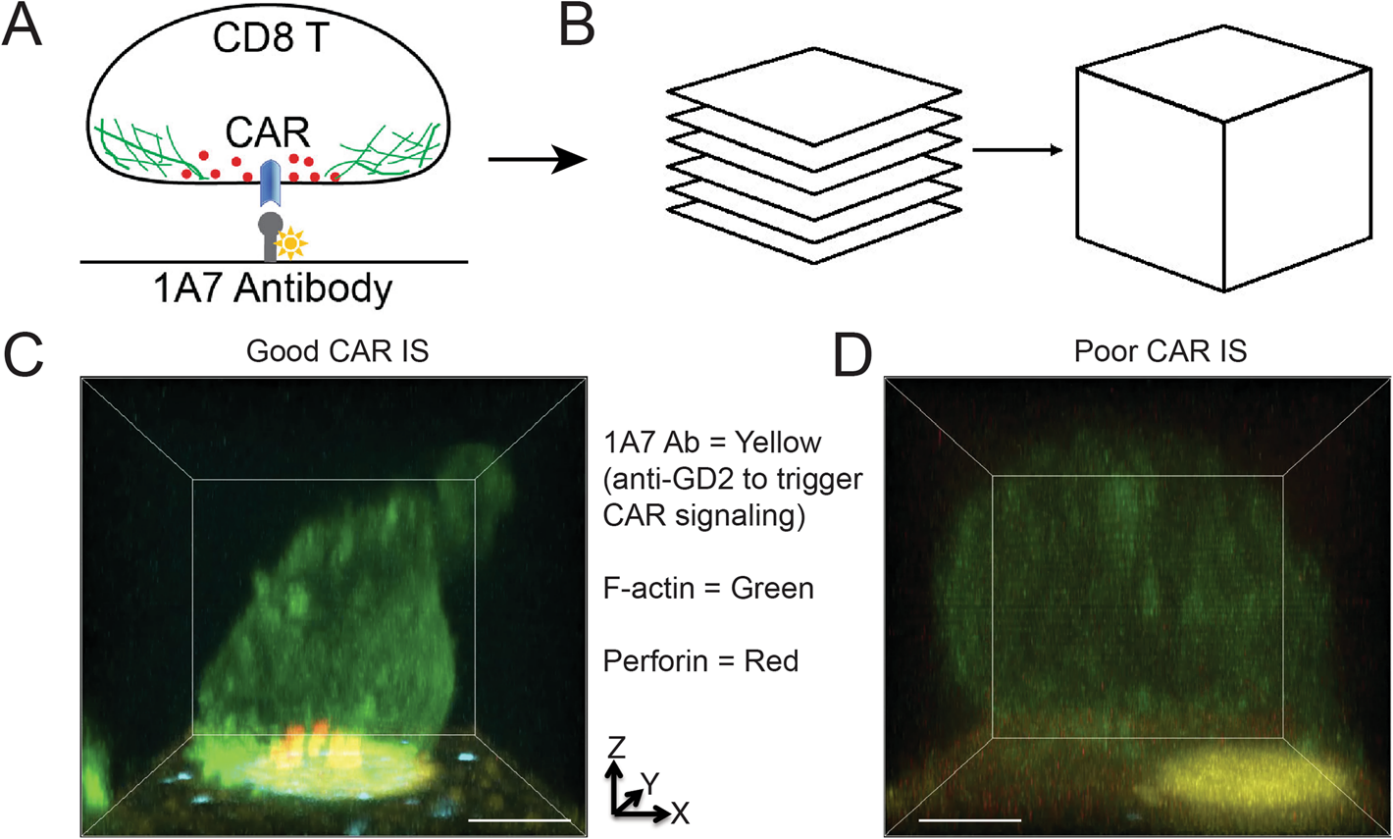
Representative CAR good and poor IS formed on the lipid bilayer. (**a**) Diagram of a lipid bilayer model to study GD2-CAR IS formation and GD2-CAR-T cell activation using idiotypic antibody (clone, 1A7) against the GD2-CAR to trigger CAR signaling on the glass-supported planar lipid bilayer system. (**b**) Schematic representation of three-dimensional (3D) confocal image reconstitutuin. Briefly, primary GD2-CAR-T cells were stimulated on the SLB containing biotinylated fluorescently labeled anti-GD2-CAR (yellow), fixed, permeabilized, and then stained with phalloidin (blue), anti-F-actin (green), and perforin (red). An individual cell was imaged under the 3D Olympus confocal setting, and then the reconstitution of these 3D confocal images . (**c** & **d**) Comparison of good CAR IS (**c**) and poor CAR IS (**d**) is illustrated by these reconstituted 3D confocal images. The x-y focal plane represents the lipid bilayer surface. The Z focal plane represents the CAR T cell position on the top of lipid bilayer. Scale bars, 5μm. Notably, stronger F-actin accumulation, lytic granule polarization, and CAR molecular accumulation is associated with optimal CAR IS

As noted above, IS quality is defined as a communication between an immune cell and a tumor cells and can be quantified by a combination of parameters that include a microscopic readouts that assess IS structure and function, as well as signal transduction outcomes [47, 63]. Methodologically, IS quality can be classified as live cell IS and fixed cell IS. Live cell IS can be qualified over a period of time. Particularly, dynamics of IS quality can be evaluated by the glass-supported planar lipid bilayer system or vertical cell pairing (VCP) system to quantify mean fluorescence intensity (MFI) of F-actin (a cytoskeleton structural molecule required for IS reorganization and stability), clustering of tumor antigen (an initiator for CAR signaling), polarization of lytic granules (LG, a maker for perforin and granzymes), and distribution of key signaling molecules (e.g., pZeta chain, a critical signaling molecule after CAR molecule microclusters) within IS as a function of time [64]. For fixed cell IS, primary human peripheral blood mononuclear cells (PBMCs) can be engineered to express CAR molecules against a particular tumor cell membrane antigen, such as the GD2 disialoganglioside (GD2.CAR), which is highly expressed in neuroblastoma (NB) [18, 65, 66]. These CAR-T cells can be stimulated by the lipid bilayer system containing molecules (e.g., Abs or tumor antigens) to trigger CAR signaling, and fixed at a particular time point. An example of good IS quality can be simply defined as: higher percentage of F-actin accumulation, stronger lytic granule polarization, and stronger key signaling molecule polarization within the IS, compared to a poor IS (Fig. 1), which is illustrated by GD2-CAR by using anti-GD2 antibody (clone, 1A7) [17],

For the lipid bilayer component, it is possible to evaluate CAR-mediated immune cell IS quality in a high-throughput, automated manner by quantitation of F-actin, clustering of tumor antigen, polarization of lytic granules (LGs), and distribution of key signaling molecules within the IS, as well as other parameters [47]. Although bilayers are artificial membranes lacking cytoskeleton, lipid rafts, and other ligands that tumor cells possess physiologically [67], they recapitulate important features such as the mobility and orientation of ligands. This allows the bilayer system to serve as a “reductionist approach” for dissecting the contribution of individual receptor and ligand (e.g., CAR molecule and its corresponding tumor antigen) in a high-throughput manner. Combination of the lipid bilayer system with other complementary assays (such as VCP device) can be employed to analyze IS structure, function, and signaling of CAR-T cells with actual tumor cells (either as tumor cell lines tumor cells isolation from patients) [64]. This latter parameter allows a robust and preclinical utility to IS quality in personalized medicine. Indeed, our preliminary studies show that IS quality measured by both the lipid bilayer system and VCP system correlates positively with the CAR cell efficacy in two different CAR-T and CAR-NK cells that share identical antigen specificity in both cell based and xenograft models [47].

We anticipate that future studies that link IS quality with other more robust quantitative outcomes (e.g., RNA-Sequence technology) will substantiate the utility of this approach. For example, recently, RNA-sequencing (RNA-seq) was recently used to identify (determinants of) response and resistance to CD19-CAR-T cell therapy of chronic lymphocytic leukemia (CLL) [49]. Transcriptomic profiling has been used comparing CAR-T cells from complete-responding patients with CLL versus non-responders showed enrichment in memory-related genes, including IL-6/STAT-3 signatures, whereas T cells from non-responders upregulated programs involved in effector differentiation, glycolysis, exhaustion, and apoptosis [49]. Such markers can be compared with IS quality parameters to assess co-variance and associated outcomes further validating IS quality as a predictive approach. Also, IS quality can be compared with other CAR associated variables such as vector integration sites and genetic backgrounds of the patients. Recent studies reported that CD19-CAR vector integration (within the host TET2 gene) was associated with CLL remission and integration-site distributions was linked to treatment outcomes [68, 69]. Potential problems with using CAR-T RNA-seq alone and insertional mutagenesis alone to predict efficacy includes limited focus on the intrinsic potency of CAR-T, which in turn precludes consideration of other factors such as the TME, the distinct genetic background of each individual patient and the differences in tumor burden from patient to patient, encounters with suppressive factors, and cell culture techniques.

In the following sections, we highlight scenario’s where IS quality might be impactful in immune-oncology applications. These applications include: (i) selection of optimal CAR products from different vendors for personalized medicine, (ii) optimization of CAR structure and design for preclinical studies or clinical trials, (iii) selection of responders of a universal CAR in clinical trials, and (iv) predicting initial responders to CAR immunotherapy, as well as predicting likelihood for relapse, as detailed below.

## Applications of IS Quality to Optimize CAR-modified Immunotherapy

### Selection of CARS from different vendors CAR for a particular patient

According to the MIT NEWDIGS Research Brief (https://newdigs.mit.edu/sites/default/files/FoCUS%20Research%20Brief%202018F210v027.pdf), there will be ~ 40-60 new cell and gene therapy CAR products approved by the FDA over the next 10 years, and while many will be designed to target the same tumor antigen, they will be derived from different commercial sources with intrinsically subtle and distinct properties. Therefore, it will be become increasingly important to prioritize which CAR will be most beneficial to a given patient. A good example of such dichotomy are the CD19-CAR-T products, whereby Novartis (Kymriah) has recently approved CAR-Ts for Acute Lymphoblastic Leukemia (ALL), and soon to be approved to treat Non-Hodgkin’s lymphoma (NHL), while Gilead CD19-CAR-T cell products (Yescarta) are approved to treat relapsed or refractory diffuse large B-cell lymphoma (DLBCL) and NHL (after two or more than 2 lines of systemic therapy), and soon to approved to treat ALL. In the future, many additional companies are poised to join in this space.

Indeed, due to the complexity of CAR-Ts compared to traditional drugs (for example, the number of atoms in a molecule of aspirin (a classic traditional medicine) is 21; however, the number of atoms in one single CD19-CAR-T cell is above 10^14^ [100 trillion]), surrogate assays such as IS quality that address structure-activity measurements will likely be required to provide information on the best efficacy for a particular patient.

**Fig. 2:**
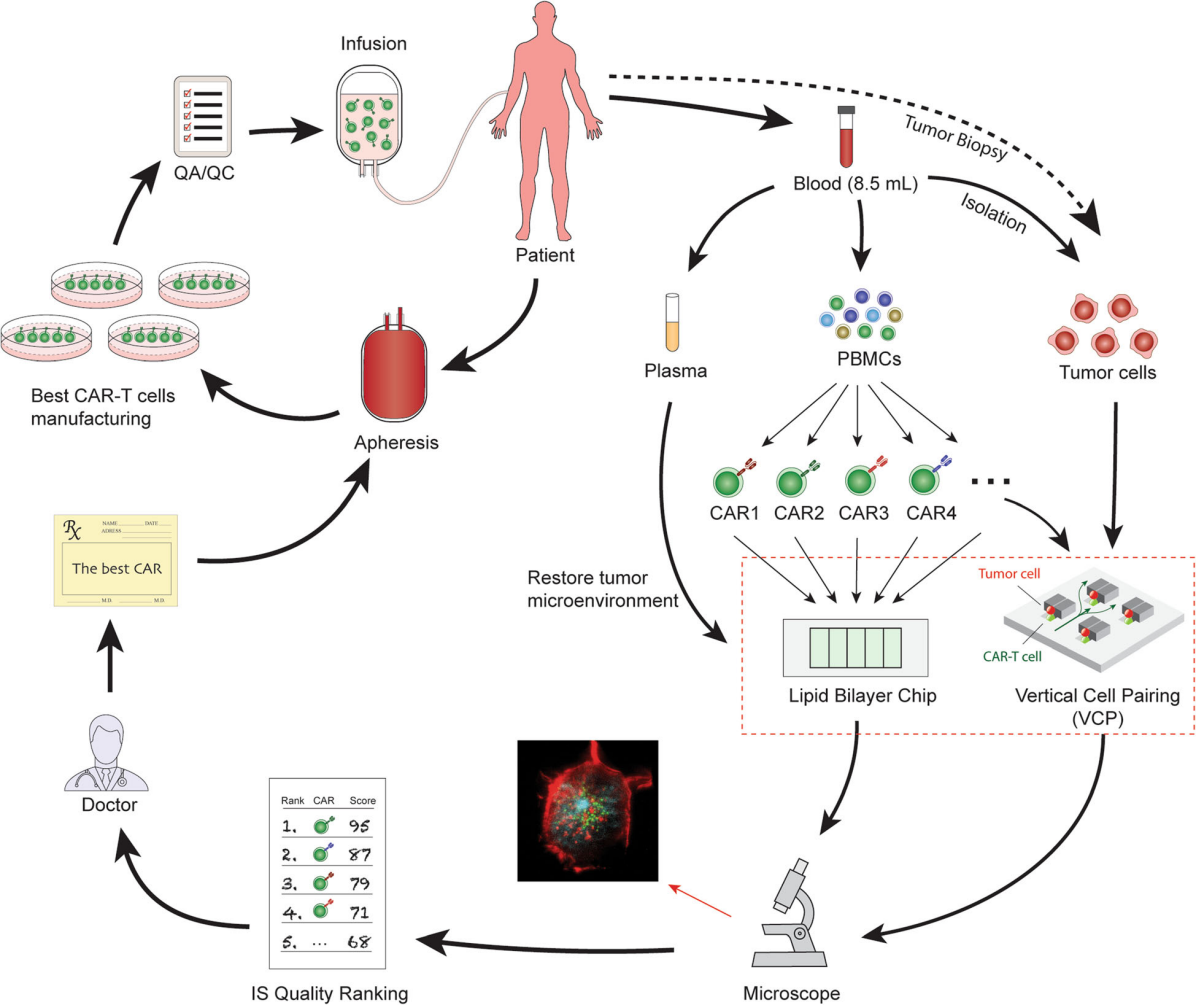
Select the best CAR from different company products for a particular patient. Blood is collected by a lab professional into a 10 ml tube with anticoagulant as a regular specimen collection procedure in any certified blood testing laboratory. The tube can be sent directly to the synapse testing lab without transferring to a secondary tube. PBMCs, plasma, and tumor cells can be enriched. Plasma can be used later for mimicking tumor microenvironment by adding the patient’s plasma to the imaging system on both the lipid bilayer system and VCP system. Meanwhile, the enriched PBMCs are placed in culture and expanded. The viral vectors containing CAR1, CAR2, CAR3, CAR4, etc. are added to generate different versions of CAR products. These different CAR products are subjected to IS quality testing. Both the lipid bilayer chip and VCP device can be used to evaluate IS quality. IS quality ranking reports can be presented to physicians who can use the data to prescribe the best CAR product for a particular patient. A manufacturing company then generates this prescribed CAR with proper quality control release testing and quality assurance review. The final product is cryopreserved and delivered to distant infusion sites, where the CAR T medicine is infused

Physicians and insurance companies will also need a novel, easy-to-use, cost-effective tool to determine which company’s CAR product will produce the best efficacy for a particular patient. Fig. 2 shows an example of how the IS quality assay can be used to select the best CAR product for a particular patient. As indicated, we have demonstrated that CAR-T cells could form a unique, functional IS on both lipid bilayers and in VCP system [47, 64]. We propose to use a novel, high-throughput microfluidics VCP system to image the IS between CAR-T cells and target cells in a vertical orientation [64]. This minimizes distortion artifacts of horizontal cell-cell conjugate imaging and provides “real life” confirmation of our reductionist, glass-supported planar lipid bilayer experiments [47]. Combining the glass-supported planar lipid bilayer system with the VCP system will provide unprecedented characterization of IS quality [47].

### Optimizing CAR design

**Fig. 3:**
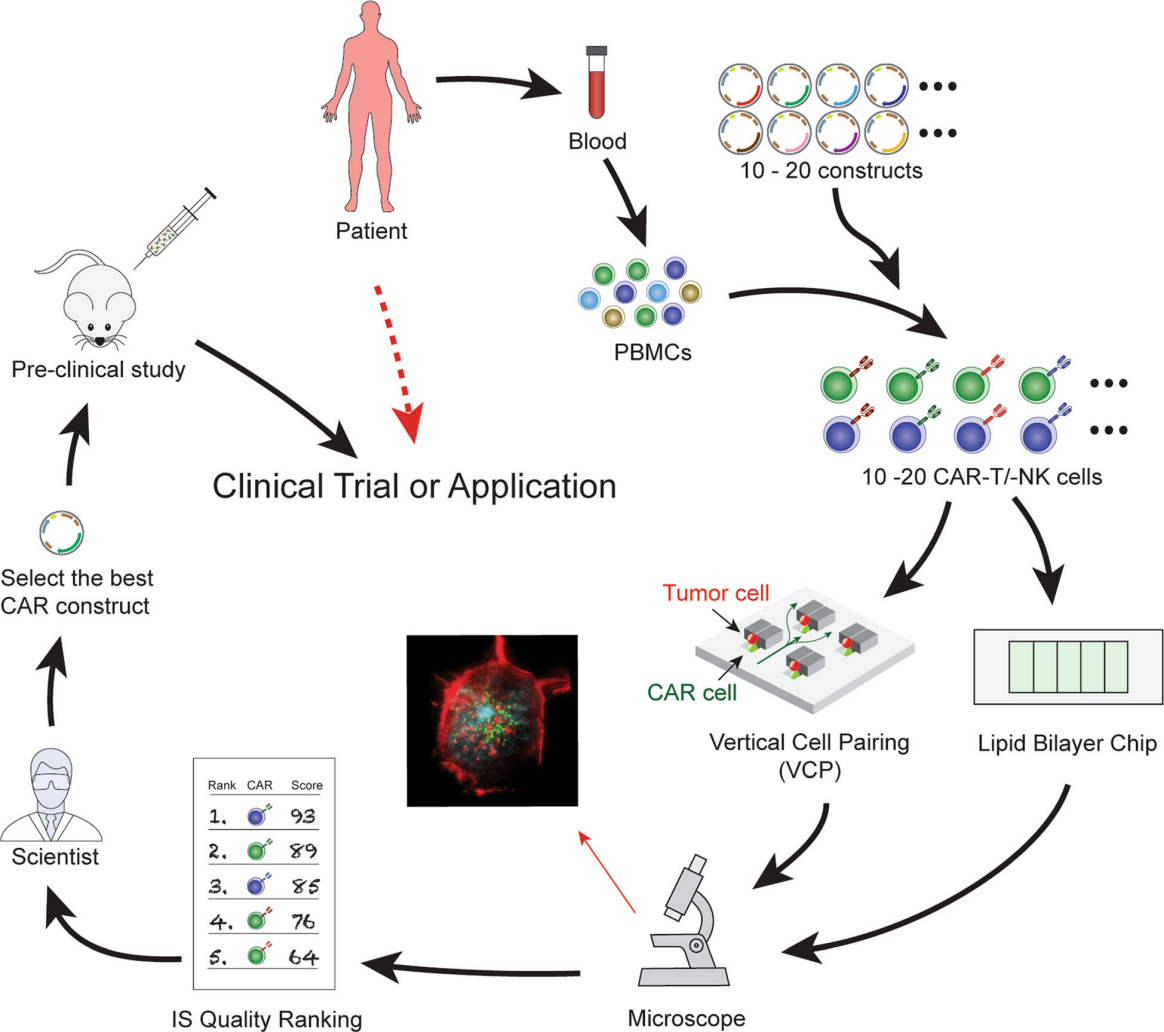
Optimizing CAR design for translational research. Blood is collected by a lab professional into a 10 ml tube with anticoagulant as a regular specimen collection procedure in any certified blood testing laboratory. The tube can be sent directly to the IS testing lab without transferring to a secondary tube. PBMCs can be enriched. The enriched PBMCs are placed in culture and expanded *in vitro*. The viral vectors containing CAR1, CAR2, CAR3, etc. are added to generate different version of CAR constructs generated by a research laboratory. These different CAR constructs are optimized to enhance the functions of CAR T. However, it is impractical to put every single construct into a preclinical study (e.g., *in vivo* animal model). The CAR T cells are subjected to IS quality testing using both the lipid bilayer chip and VCP device. IS quality ranking reports can be presented to scientists who can select the best CAR construct for a preclinical animal study, and ultimately, the optimal CAR design for downstream clinical applications. The SPE system can identify the best CAR construct from numerous constructs by screening the IS quality

A second utility of IS quality in CAR screening in personized medicine will be to assess CARs with different designs and structural modifications [70]. These include , but are not limited to (i) selection of monoclonal antibodies with different affinities for the same tumor antigen, (ii) selection of different size linkers, and (iii) the use of different extracellular or intracellular domains of co-stimulatory molecules [25]. For example, different biotech companies use different clonotypes of CD19 antibodies in producing the CD19-CAR-T cells. These clonotypes consist of either an identical extracellular-domain with different intracellular domains or an identical intracellular-domain with different extracellular domains, all of which can influence the functional utility of CARs. As shown in Fig. 3, IS quality assay can rapidly rank the predicted effectiveness of a variety of CAR constructs with minor modifications (for example different constructs of CD19-CAR are generated from different institutes or companies, as well as research laboratories). The rapidity and usability of the IS quality assay not only confers significant advantages over conventional assays but has broad potential applications (e.g., development of CDx [companion diagnostics] device in clinic) for researchers to quickly ascertain which constructs have the optimized clinical value/outcome(s).

### Selecting responders to a universal CAR-modified cell in clinical trials

**Fig. 4:**
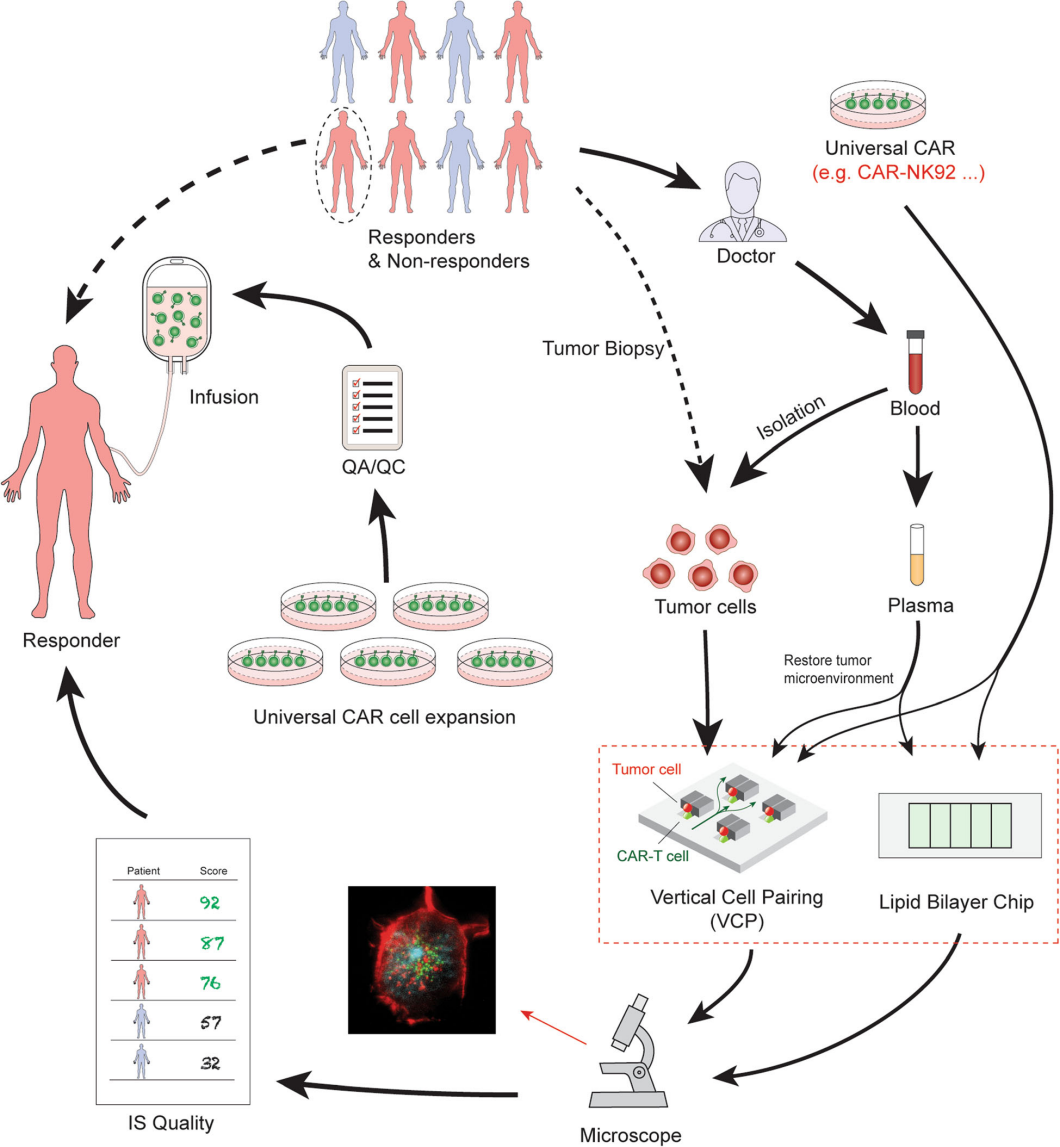
Select patients who will respond to treatment with universal CAR-modified cells or a particular CAR product. To select responders for a particular CAR treatment, tumor cells or plasma can be isolated from 8 ml of peripheral blood from each patient. CAR cell IS quality can be assessed by lipid bilayer and VCP device. Briefly, blood is collected into a 10 ml tube with anticoagulant as a regular specimen collection procedure. The tube can be sent directly to the synapse testing lab without transferring to a secondary tube. PBMCs, plasma, and tumor cells can be enriched. Plasma can be used later for mimicking the tumor microenvironment by adding the patient’s plasma to the imaging system on both the lipid bilayer system and VCP system. Meanwhile, the enriched PBMCs are placed in culture and expanded. The viral vector containing the universal CAR construct such as CD19-CAR, is added to generate CAR products. These universal CAR products are subjected to IS quality testing. Both the lipid bilayer chip and VCP device can be used to evaluate the IS quality of universal CAR products in response to tumor cells isolated from each individual patient. IS quality ranking reports can be presented to physicians who will base their decision of which CAR product to prescribe. The informed physicians can select a particular patient (responder) for a particular CAR treatment or clinical trial. Meanwhile, for non-responders, IS testing can identify another suitable CAR therapy. The manufacturing company generates the prescribed CAR for this patient with proper quality control release testing and quality assurance review. The final product is cryopreserved and delivered to infusion sites, where the CAR T medicine is infused

A third utility concerning IS quality in personalized medicine and immunotherapy will attempt to ascertain whether a single ‘off-the-shelf’ universal CAR product can be effectively developed [71], and if so, which selected patients it will most benefit. Clearly, while the generation of a universal, standardized ‘off-the-shelf’ CAR-modified T cell product is conceptually meritorious and would significantly reduce the cost of immunotherapy, the ability to achieve global anti-tumor ability of universal CAR-modified cells *in vivo* is complex and multifactorial. There are multiple factors that contributes to the clinical responses that include (i) the intrinsic potency of CAR factors (e.g., different subsets of CAR-T cells, etc.), (ii) tumor-specific factors (e.g., mutations of tumors and inhibitory ligand expressions, etc.), (iii) patient-to-patient variability (e.g., age, sex, disease stages, exercise frequency, etc.), and (iv) tumor microenvironment factors (e.g., cytokine milieu and metabolism, etc.). Although the intrinsic potency of CAR factors is, a priori, expected to be identical in the scenario of a universal CAR cells used, aforementioned tumor factors, patient-to-patient variability, and tumor microenvironment will all impinge on efficacy. Due to these complexities, while presently no single parameter can predict a patient’s clinical outcome in the CAR-T therapy, we predict that IS quality can be utilized to select responders to a universal CAR-modified cells (as a go/no-go to enroll in clinical trials), and that IS quality assays might yield clear differences in IS structure, function, and signaling between responders and non-responders (patient-to-patient variability) by first isolating tumor cells from patients followed by incubation *in vitro* with the universal CAR and assessment of IS quality (Fig. 4). As such, a “personalized IS quality” (i.e. the quality of IS formed between universal CAR-T cells and the susceptible of a range of tumor cells from an individual patient) could be envisioned in conjunction with other markers used in clinical practice as a composite clinical predictor. Such IS quality technology can be used to allow a clinically applicable, high-throughput test.

### Predicting initial responders to CAR immunotherapy as well as predicting likelihood of relapse

A final goal and utility of IS quality and utility will be to assess dynamically whether a patient will initially respond to a given first-in-class CAR immunotherapy, and subsequently, if and when a patient is most prone to relapse or remain in remission longitudinally [28, 72]. For example, to assess initial responsivity, a specific CAR-modified immune cells could be incubated with a patient’s own tumor cells for IS quality as outlined above. For relapse/remission longitudinal studies, after initial CAR infusion, patient’s blood samples can be collected to evaluate IS quality to determine if IS quality changes over time in a dynamic and temporal fashion. In the case of predicting initial responsivity, a recent clinical trial (ClinicalTrials.gov NCT00924326) demonstrated that while clinical remissions of lymphoma are associated with elevated serum levels of IL-15, and to a lesser extent IL-10 [48], serum levels of the majority of 39 other proteins (including cytokines, chemoattractants, adhesion molecules, etc.) were not significantly different in responders vs. non-responders [48], which indicates other better paramentes are needed.

In another clinical trial on kappa-CAR [35] and CD30-CAR [34] (ClinicalTrials.gov NCT00881920 and ClinicalTrials.gov NCT01316146), outcomes studies also show that conventional clinical measures, including percentage of CAR positive cells, white blood cell count, CD4/CD8 ratios, absolute neutrophil count, and IL-6 ratios do not correlate well with clinical response between responders and non-responder. Additionally, no close correlations between transgene levels in the peripheral blood, pre-infusion absolute lymphocyte counts, disease type, CAR cell dose, cytokines used in culture (IL-2 vs. IL-7 and IL-15) and clinical response [35] are noted. Clearly, better predictors of clinical outcome are required. IS quality evaluation may provide a tool for determinizing patients who will or will not respond to a particular CAR product in clinic (Fig. 4).

**Fig. 5:**
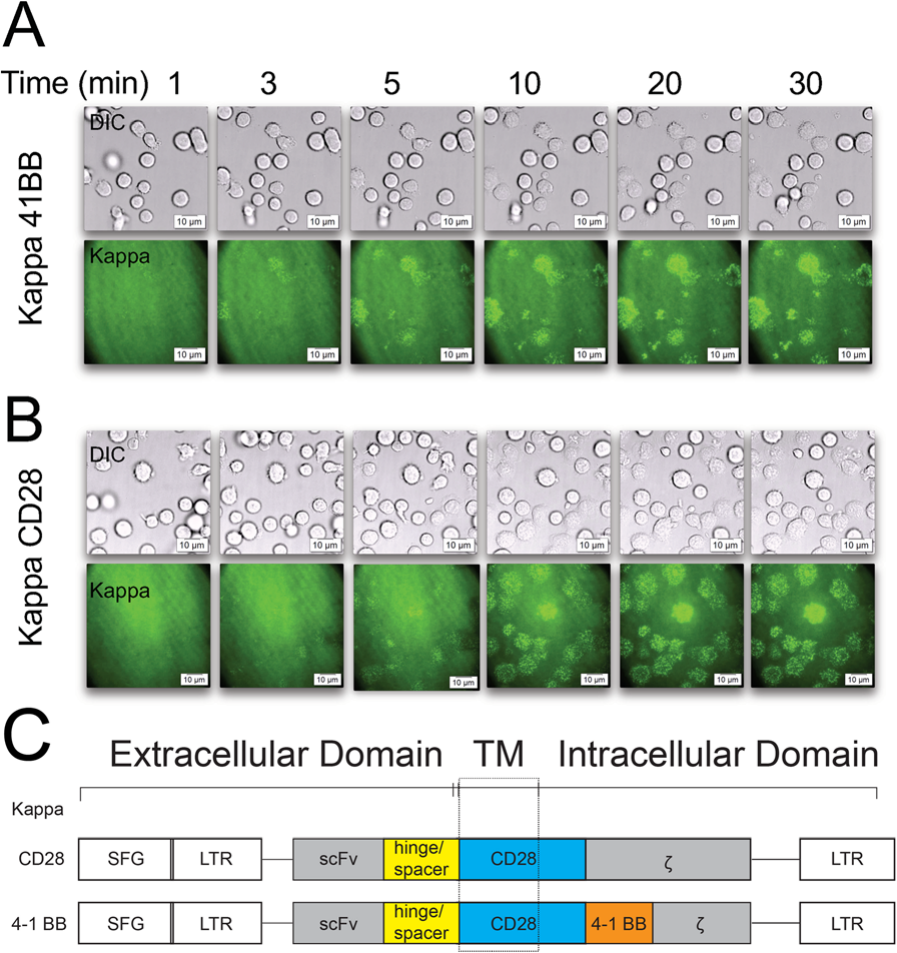
Kinetics of IS formation in live Kappa-CAR T cells under total internal reflection fluorescence microscopy (TIRF) to evaluate the dynamics of CAR IS quality. All cells were imaged by TIRF microscopy on lipid bilayers carrying Kappa protein conjugated with Alexa Fluor 488 at the indicated time points. **a** Kappa-41BB cells were imaged at 1 min, 3 min, 5 min, 10 min, 20 min, and 30 min. Time-lapsed differential interference contrast (DIC) (top panel) and TIRF (bottom panel) images are shown. **b** Kappa-CD28 cells were imaged at 1min, 3min, 5min, 10min, 20min, and 30min. Time-lapsed DIC (top panel) and TIRF (bottom panel) images are shown. **c** Schematic representation of recombinant retroviral vectors encoding kappa-CAR. Both Kappa-CD28-CAR and Kappa-4-1BB-CAR constructs (CD28 and 4-1BB) contain CD28 transmembrane domain and intracellular domain of CD3 zeta. The difference between Kappa-CD28-CAR and Kappa-4-1BB-CAR constructs is that Kappa-4-1BB-CAR construct contains intracellular domain of 4-1BB molecule

Finally, since CAR-T cells are considered ‘living’ drugs that differ from conventional medicines (such as chemical compounds, nucleic acids, or proteins) there are several caveats for CAR-T biology that must be considered that include; (i) a subset of memory-like CAR-modified T cells can live for decades in patients’ bodies [49]; (ii) CAR modified T cells can reside in the bone marrow, when the tumor antigen has been eradicated [9], and (iii) CAR-modified T cells can divide and proliferate upon tumor antigen stimulation. We anticipate that our current work brings significant conceptual and technical innovation to improving our understanding of CAR-T cell biology, with the ultimate goal of providing design guidance for CAR optimization for patients with cancer. As described above, we propose using a novel biophysical and high-resolution quantitative imaging approach to evaluate CAR-T cell IS quality. This will provide an easy-to-use predictor of efficacy against both hematologic (e.g., leukemia and lymphomas) and solid (e.g., neuroblastoma) tumors. For example, CAR-modified cells can be visualized forming an IS on a glass-supported lipid bilayer in real-time (Fig. 5) or on a vertical cell pairing device with patient’s own tumor cells [64]. TIRF (total internal reflection fluorescence) microscopy can be utilized to visually observe and measure the kinetics of IS formation and quality, making it an extremely useful tool to predict the efficacy of any given CAR product. As a proof-of-concept to test this hypothesis, we chose kappa-CD28-CAR and kappa-4-1BB-CAR constructs because these two types of constructs we used are representative 2^nd^- and 3^rd^- generation of CAR constructs that currently used in clinical trials by Baylor College of Medicine (BCM) at the Center for Cell and Gene Therapy, which allows us to collect the clinical samples in follow-up studies in the future. Given the unprecedented pace of advances and challenges in immunotherapy for cancer, it is imperative that we are able to rapidly and uniformly rank the efficacy of various CARs with different optimizations from different investigators and pharmaceutical companies. Our assay for IS quality assessment is expected to fulfill many of these urgent needs.

## Current Challenges in CAR-modified Immune Cell Therapy

### Significant Toxicity of CAR-T Cell Immunotherapy

The above discussions have focused on a conceptual idea that IS quality can better predict CAR efficacies in engineered CAR cells. Ultimately, we also posit that IS quality may also have predictive value in assessing and predicting toxicities and side effects commonly observed with CAR-T biology *in vivo*. Clearly, a standardized pre-clinical assay(s) that might predict subsequent toxicities could potentially reduce life-threatening toxicities [36–40] and deaths [39, 73, 74]. Common target effects of include, tumor lysis syndrome, gastrointestinal bleeding and perforation, myelosuppression-related infections, cytokine release syndrome (CRS), anaphylaxis, graft versus host disease (GvHD), allergy, and autoimmunity caused by T cell products [36, 75] and CAR-related neurologic events [15, 36]. However, generally, common toxic side effects of CAR-modified immune cells can be broadly divided into the following categories: CRS, neurotoxicity, on-target and off-tumor effects of CAR-T cells, cardiotoxicity, and hypersensitivity reactions to CAR-modified cells [15, 36, 76]. Predicting co-variance in the side effects and toxicities with IS quality may have therapeutic potential in cancer immune-oncology. Below, we summarize major toxicities of CAR and potential mechanisms.

#### Cytokine release syndrome (CRS)

The most common toxicity of CAR-modified immune cells is CRS, although exact molecular mechanisms of CRS are not fully understood. CRS is defined as an excessive cytokine release (IL-1, IL-6, IFN-γ, and IL-10) by CAR-modified immune cells or bystander innate immune cells (e.g., macrophages, monocytes, dendritic cells, and other immune cells). These excess cytokines (driven mainly by IL-6) can cause vascular leakage with associated respiratory failure, coagulopathy, and multi-organ system dysfunction [77]. One potential mechanism for CRS is that CAR-T activated cells produce a large amount of interferon gamma (IFN-γ) and/or tumor necrosis factor alpha (TNF-α) that subsequently activates macrophages, dendritic cells, and endothelial cells. These cells activated by IFN-γ or TNF-α can further release proinflammatory cytokines (e.g., IL-6). Excess IL-6 produced by activated macrophages and endothelial cells provide positive feedback to further activate CAR-T cells and other immune cells, leading to CRS [78, 79]. In addition to IL-6 [15, 80], recent studies show that IL-1 also plays an important role in CRS [81, 82], whereby an IL-1 receptor antagonist, (Anakinra) can effectively control CRS and neurotoxicity in humanized mice [81]. This finding requires further testing in clinical trials. Additionally, recent studies have shown that myeloid-derived catecholamines are essential mediator for CRS in MTR (metyrosine) and ANP (atrial natriuretic peptide) mouse model [83]. Importantly, the FDA-approved catecholamine synthesis blocker metyrosine (Demser) can reduce the CRS without impairing the CD19-CAR-T cell therapeutic response. Compared to tocolizumab (a humanized monoclonal antibody against the interleukin-6 receptor), Demser is much inexpensive to manufacture, however, the effects of Demser require further testing in clinical trials.

#### Neurotoxicty

Neurotoxicity is also a potentially life-threatening toxicity in patients treated with CAR-T cell therapy, but etiologically, also not completely understood. Symptoms broadly include hallucinations, encephalopathy, seizures, aphasia, headaches and most seriously, rapidly progressive cerebral edema [84]. The incidence of neurotoxicity is variable in patients (0 - 50%) [13–15, 85–89], that in part depends on the different types of CAR constructs used, and can occur in either the presence or absence of CRS. A number of studies have shown trafficking of CAR cells in the central nervous system (CNS) or cerebrospinal fluid of patients who experience neurotoxicity, but not in brain areas [13, 14, 90] where it is thought that cytokine-mediated inflammation also contributes to neurotoxicity [91].

In addition to the direct attack of CAR-T cells on the CNS, patients with severe neurotoxicity showed evidence of endothelial activation, including disseminated intravascular coagulation, capillary leak and increased blood–brain barrier (BBB) permeability [39]. In the brains of mice experiencing CRS, damage to the intestinal-epithelial barrier and subsequent infiltration of T cells was detected, suggesting yet another potential mechanism of neurotoxicity [84, 92, 93].

#### Cardiotoxicty

Clinically, cardiovascular manifestations of CRS include hypotension, troponinemia, arrhythmias (including tachycardia), decreased left ventricular systolic ejection fraction, and QT prolongation [13–15]. The reduction in cardiac output associated with CRS is similar to that of stress-induced cardiomyopathy seen in sepsis, but specific pathophysiologies remain unclear [94, 95]. Cardiac arrest can occur seven days after CAR-T cell infusion in patients with acute lymphoblastic leukemia (ALL). There have also been reports of a left ventricular ejection fraction that was 25% of the normal baseline [8, 87, 96–98].

#### On-target/off-tumor toxicties

“On-target/off-tumor” toxicity, in the form of chronic B cell aplasia, is considered a tolerable side effect of this therapy as it can be treated with intravenous immunoglobulin (IVIG) replacement therapy [99, 100]. Additionally, hypersensitivity reactions (anaphylaxis or allergy) to CAR-modified cells can be related to host recognition of infused foreign antigen because the majority of CAR-T cells contain mouse monoclonal antibody (mAb). When multiple infusions of CAR-T cells are applied, the allergy reaction occurs. Using humanized antibody in CAR design is important in reducing hypersensitive reactions to CAR-T cells [36].

Finally, in addition to significant toxicities and comorbidities by infused CARs, there is significant tumor relapse after CAR-mediated immunotherapy [101, 102]. Multiple clinical trials (e.g., ClinicalTrials.gov numbers, NCT01626495, NCT01029366 and NCT02208362) have demonstrated that the cause or potential cause of tumor relapse in CAR-T cell immunotherapy targeting CD19 and IL-13Rα in patients with ALL and glioblastoma is the loss of antigen [13, 103]. Among these world’s leading academic institutions on cell and gene therapy, including Memorial Sloan Kettering Cancer Center (ClinicalTrials.gov number NCT01044069) on B Cell ALL [15, 77, 104], Baylor College of Medicine (ClinicalTrials.gov number NCT00840853) [105], National Cancer Institute (ClinicalTrials.gov number NCT01593696) [14], Fred Hutchinson Cancer Research Center (ClinicalTrials.gov numbers NCT02028455 and NCT01865617), several clinical trials show different cases of relapse in these complete remission patients [85]. According to these and other reports from other countries, tumor relapse after CAR-mediated immunotherapy is significant. In addition to tumor antigen loss and mutations [106, 107], the precise mechanisms for relapse after CAR therapy remain elusive [108].

Collectively, there is a great unmet need to predict and reduce potentail toxicities from CAR products. We posit that features of the IS quality synapse can be useful to predict certain toxicties of CAR products mentioned above. Further studies that compare responders with IS quality are required to substantiate this research field.

### Challenges of CAR immunotherapy in solid tumors

A further consideration will be to utilize IS quality to predict the success of novel tumor associated antigens identified on solid cancer cells. As noted above, while CAR-T immunotherapy has been remarkably successful in treating hematological malignancies, CARs have limited effects in solid tumors and face many challenges in solid tumor CAR-T clinical trials [109]. Identifying a unique tumor-associated antigen (TAA) in most cancers remains the primary challenge to employing CAR-T immunotherapy for treatment of solid tumors. Ideally, tumor-restricted antigens should mainly be expressed on the tumor cell surface and exclusively expressed in tumors but not in healthy tissues to avoid potential immune reactions against healthy tissues due to “on-target/off-tumor” toxicity [70, 110–115]. Nearly 30 types of cell surface antigens that are highly expressed by solid tumors are currently being investigated as targets for CAR-T cell therapy [113, 116]. However, none of them are expressed exclusively on tumor (including both liquid and solid tumors) cell plasma membranes.

The local tumor microenvironment (TME) poses another important challenge to CAR-T cell immunotherapy in solid tumors [117], as well as the IS quality. Various physical and environmental barriers (e.g., extracellular matrix) that surround solid tumors are not present in liquid cancers. Thus, CAR-T cells have difficulty in locating to and accessing the solid tumors and insufficient penetrance of CAR-T cells into solid tumor tissue remains a major impediment to this research field [118]. Additionally, chemokines uniquely secreted in the TME of solid tumors, such as CXCL12, can engage CXCR4 on the CAR-T cell surface and inhibit T-cell migration into the tumor [119, 120]. Endothelin B receptor (ETBR) on the endothelium of blood vessels within the tumor reduces T-cell adhesion and compromises their ability to extravasate [121]. The TME also impairs the trafficking and infiltration of CAR-T cells into solid tumors through other potential mechanisms, such as the CCR2/CCL2 axis, regulatory subunit I anchoring disrupter (RIAD) peptide, extracellular matrix proteins, oxidative stress, nutrient starvation, low pH and hypoxia [114, 122–125]. In addition to these ‘chemical exclusion’of CAR-T cells from tumor beds by these chemokines and receptor/ligand interactions, the ‘physical exclusion/barriers’ of CAR-T cells from tumor beds by collagen fiber bundles or extracellular matrix deposition may add another layer of complex of CAR-T cell trafficking and infiltration into solid tumors [26, 126].

Additioanlly, immune checkpoint molecules can also affect CAR-T immunotherapy [127–129]. While blockades via the programmed cell death protein-1 (PD-1) receptor and cytotoxic T-lymphocyte antigen 4 (CTLA-4) have led to breakthroughs in cancer immunotherapy [130], interestingly, CAR-T cells also express PD-1 and CTLA-4, and others [131]. For examples, recent studies show that exhausted CAR-T cells express other inhibitory immune checkpoint receptors, such as T-cell immunoglobulin and mucin-domain containing-3 (TIM-3) [130]. The immune inhibition induced by PD-1 and other inhibitory receptors can be exploited by a variety of tumor and stromal cells expressing the programmed cell death ligands (e.g., PD-L1 and PD-L2) or CTLA-4 [132–134]. Recent studies show that modifying CAR-T cell to secrete PD-1-blocking single-chain variable fragments (scFv) [135] or replace PD-1 inhibitory signaling domain with CD28 activating domain [136] can enhance the efficacy of CAR-T cells by modulating the TME. However, other studies also show PD-1 knockdown impairs in vivo persistence and proliferation of CAR-T cells [137]. Presently, the optimal way to modulate PD-1 signaling in CAR-T remains incompletely understood.

The anti-inflammatory and pro-inflammatory cytokine environment also play an important role in regulating CAR-T functions [138, 139]. These factors may also affect the IS quality. Suppressive immune cells in the TME often preferably express pro-tumor Th2 secreted cytokines, such as IL-4 and IL-13, rather than anti-tumor Th1 (e.g., IL-2 production), or secreted cytokines like IFN-γ and tumor necrosis factor-β (TNF-β) [140, 141]. Cytotoxic T-cell functions are inhibited by cytokines such as IL-4, IL-10 and transforming growth factor (TGF)-β, which are secreted by suppressive immune cells in the TME such as regulatory T (Treg) cells, myeloid-derived suppressor cells, and tumor-associated macrophages/neutrophils. These cells are generally thought to represent a significant barrier against CAR-T cell functions [142].

Metabolism-associated immune suppression in the TME also inhibits CAR-T cell functions [143]. Cancer cells are highly metabolically active and have relatively high amounts of glycolysis and glutaminolysis [144]. These metabolic pathways result in a distinct accumulation in the TME of metabolites such as lactate, prostaglandins, cyclooxygenase (COX)-1/2, indoleamine-2,3-dioxygenase (IDO), tryptophan-2,3-dioxygenase (TDO), arginase-1 and nitric oxide synthase (NOS) [145]. The exact molecular mechanisms by which these factors may compromise CAR-T functions are the subject of emerging research in the field of immunotherapy.

### The High Cost of CAR Therapy

A final consideration is whether is whether IS quality can be used to ultimately reduce costs of CARs. The cost of CAR-based immunotherapy is currently prohibitive [41]. The “list price” for a single infusion of tisagenlecleucel (Kymriah) from Novartis (Basel, Switzerland) is $475,000. The price for axicabtagene ciloleucel (Yescarta) from Kite Pharma (a Gilead Company, Los Angeles, CA) is $373,000 [146]. One estimate has the cost as high as $1.2 million per injection. Together, with the variety of CAR constructs already available and those in development, it is becoming imperative to predict which construct is likely to work best for an individual patient prior to embarking on treatment, which is also crucial to clinical trials. The field has been divided into two pathways to reduce the high cost of CAR therapy. The first pathway is to generate the universal, ‘off-the-shelf’ CAR [147], which includes CAR-T cells with MHC, TCR deletion, or antigen-targeting domain split [148]. The second pathway has been focused on NK cells [63, 149, 150], NKT [151], and γδT cells [71]. Clearly, due to the high cost of CAR therapy, it is imperative to develop surrogate tools to predict the efficacy and toxicity of CAR products.

## Perspectives

**Fig. 6:**
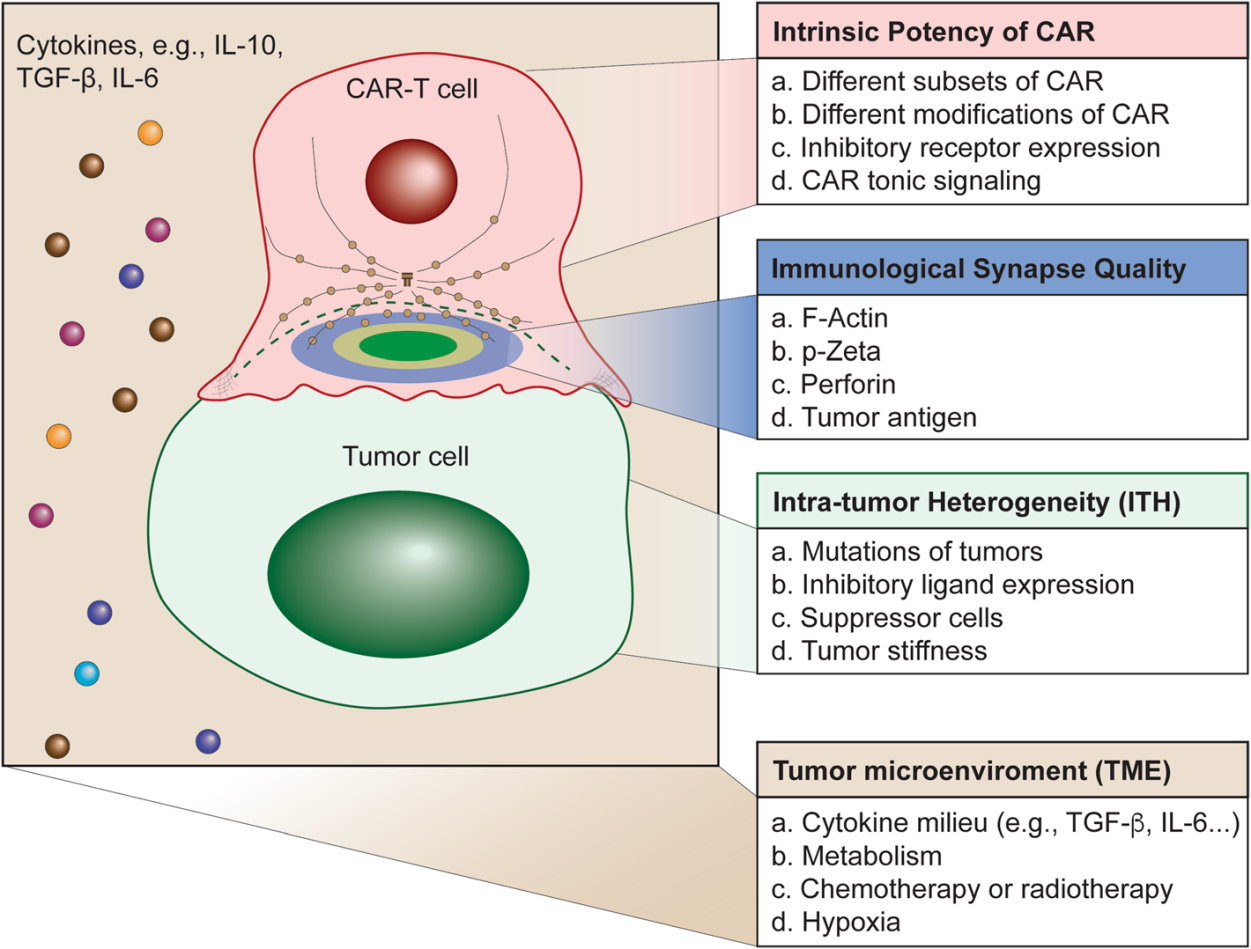
Potential Multiple Factors Determine the Quality of Immunological Synapse in Cancer immunotherapy. The development of a novel SPE approach to predict the effectiveness of Chimeric Antigen Receptor (CAR)-modified cells by quantifying the quality of immunological synapse is dependent on multiple factors. In addition to patient conditions (e.g., age, sex, tumor burden, stage of diseases, tumor antigen mutations & loss, etc.), there are three main aspects to be considered to quantify the quality IS. First, intrinsic potency of CAR-modified immune cells includes different subsets of CAR-modified immune cells, different modifications of CAR constructs, inhibitory receptor expression, and CAR tonic signaling. Second, intra-tumor heterogeneity (IHT) includes mutations of tumors, inhibitory ligand (e.g., PD-L1) expression, suppressor cells, and tumor stiffness. Third, tumor microenvironment (TME) contains cytokine milieu, metabolites, hypoxia, and collagen fibers around tumor cells

The therapeutic value of CAR-mediated immunotherapy for hematologic malignancies and promised therapeutic value in more and more cancers, including solid tumors, has led to explosive growth and development of CAR-based immunotherapy. As developed in this review, the potential for IS quality to serve as a proxy for CAR-modified immune cell effectiveness with the ultimate goal of inducing complete remission in patients with an expanding number of cancers represents an exciting new dimension in CAR biology. The SPE system under development promises to address many challenges to the applicability of new CAR-T constructs and adoptive CAR-T cell transfer into individual patients. However, to develop SPE, additional challenges in both basic and clinical research still remain: In characterizing the basic biology of IS’s, we do not yet know:

1. Kinetics: A. What is the difference in immunological synapse dynamics between CAR and T cell receptors (TCR)? B. What is the activation threshold for CAR cells on the level of a single IS? How many molecules of CAR per IS are sufficient to fully activate a CAR-T or CAR-NK cell? How does the IS quality contribute to signal strength? Is the IS quality a cause or an effect of CAR signaling strength? C. What is the best way to quantify the dynamic IS quality over time?
2. Immune cell biology: A. What is the influence of various *in vitro* culture medium or individual plasma environments (e.g., anti-/pro-inflammatory cytokines) on culturing human CAR-T cell IS? B. What is the best way to engineer and manufacture optimum T cells without inducing exhaustion of CAR-T cell IS? One potential issue regarding CAR-T expansion *in vitro* using antibodies (e.g., anti-CD3 and anti-CD28) in the presence of cytokine (e.g., IL-2) is that the CAR-modified cells become exhausted after rapid proliferation and differentiation [152]. For example, CAR-modified immune cells express exhaustion markers such as PD-1 [153–156]. Does PD-1/PD-L1 engagement (or other inhibitory receptor signaling) change the IS quality? Does co-stimulatory molecule engagement (e.g., CD28/CD80, OX40/OX40L, etc.) change the IS quality? C. How can immunosuppressive checkpoints on the surface of CAR cells be managed? How do CAR cells develop into memory-like CAR cells on the level of IS? What is the difference between memory-like CAR-T IS and naïve CAR-T IS?
3. Translational research: A. What is the distribution and localization of CAR cell IS in peripheral organs after infusion? B. How do CAR cells pass the blood-brain barrier on the level of IS? C. What are the effects of exosomes released by tumor cells on the quality of IS formed by CAR cells? D. Which molecule within CAR IS can be used as the most effective marker for judging the IS quality? E. Does intra-tumor heterogeneity (ITH) affect the CAR-T IS quality? TMC, intrinsic potency of CAR-T, and ITH are three main factors that may determine CAR-T IS quality (Fig. 6). What is the hierarchy among these three factors?

The pressing clinical questions addressed in this review attempt to investigate the feasibility of using CAR-T cell IS quality as a marker for CAR-T effectiveness in individual patients. The proposed concept of SPE impacts public health and addresses a key immunobiological knowledge gap to define the dynamics of the CAR-T cell IS and understand the underlying molecular basis of CAR IS. The potential application of SPE can be classified into two main aspects: basic research application and clinical research application. Development of high-throughput functional screens to identify high-performance CAR-modified immune cells and continuing immunobiology of CAR research are both required to optimize CAR cell biology. Clinical research applications include development of high-throughput screens to identify responder and non-responder patient populations and development of high-throughput screens to select the best CAR for a particular patient.

## Conclusions

**Fig. 7:**
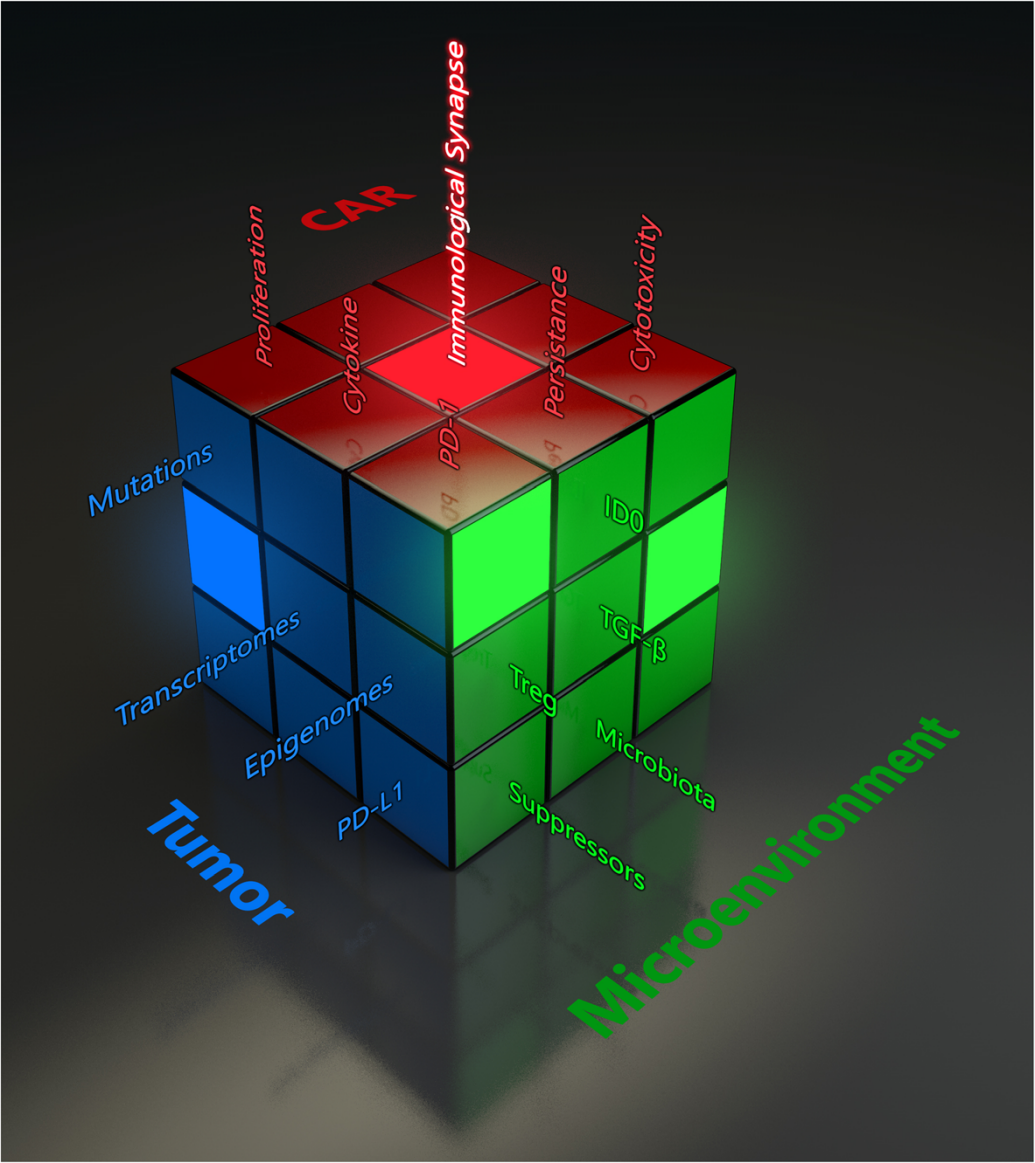
The various branches of evaluating cancer immunotherapy metaphorically represented as a Rubik’s cube. The development of a novel approach to predict the effectiveness of Chimeric Antigen Receptor (CAR)-modified cells by quantifying the quality of CAR IS will introduce a new parameter to the rapidly expanding field of cancer immunotherapy. Currently, no single parameter can predict the clinical outcome or efficacy of a specific type of CAR-modified cell. IS quality will serve as a quantifiable measure to evaluate CAR products and can be used in conjunction with other conventional parameters to form a composite clinical predictor. Much like a Rubik’s cube has countless configurations, several methods and combinations of clinical metrics have arisen for evaluating the ability of a given immunotherapeutic strategy to treat cancer. The quality of IS depicting cancer immunotherapy is metaphorically expressed as a Rubik’s cube. Each face/color represents one aspect of cancer therapy. Each grid in one face indicates one factor within that aspect of cancer therapy. For example, the green color represents the tumor microenvironment, and one out of the nine grids in the green color indicates suppressor cells (suppressors in green). Changes in one factor may completely alter the entire strategy of cancer therapy. However, the quality of IS (illuminated center red grid) makes the effectiveness of CAR immunotherapy predictable

Time is of the essence for development of this test to predict the efficacy and toxicity of CAR cells. Accurate predictors of efficacy and toxicity are required to reduce costs as well as minimize the time it takes to get the appropriate therapy to patients. While many groups are experimenting with a variety of CAR constructs, we believe IS quality is the first to investigate a uniform, concrete and patient-specific way to rank their efficacy. According to the Precision Medicine Initiative (https://ghr.nlm.nih.gov/primer/precisionmedicine/definition), precision medicine is “an emerging approach for disease treatment and prevention that takes into account individual variability in genes, environment, and lifestyle for each person.” As the “Rubik’s Cube” for evaluating cancer immunotherapy (Fig. 7) becomes more and more complex, developing a universal parameter to deliver the highest quality therapy to patients is critical. However, this SPE concept needs to be tested carefully because the attributing factors of clinical response include more than hundreds of parameters. The IS quality cannot serve as a ‘one size fits all’ role in determining the efficacy of CAR therapy. However, the concept of SPE can be used as a key parameter in conjunction with other conventional parameters to form a composite clinical predictor in the future.

Hopefully, the proposed SPE approach will tailor CAR-T cell immunotherapy to individual patients with specific cancers – saving time, money and ultimately, lives.

## Data Availability

The datasets during and/or analyzed during the current study available from the corresponding author on reasonable request.

## Abbreviations

CAR: Chimeric antigen receptor
NK: Natural kill cell
CRS: Cytokine release syndrome
CTL: Cytotoxic T lymphocytes
IS: Immunological synapse
IHT: Intra-tumor heterogeneity
TME: Tumor microenvironment
TAA: Tumor-associated antigen

## References

[1] Li F, Zhang T, Cao L, Zhang Y (2018). Chimeric Antigen Receptor T Cell Based Immunotherapy for Cancer. Curr Stem Cell Res Ther.

[2] June CH, O'Connor RS, Kawalekar OU, Ghassemi S, Milone MC (2018). CAR T cell immunotherapy for human cancer. Science.

[3] Ribas A, Wolchok JD (2018). Cancer immunotherapy using checkpoint blockade. Science.

[4] Gross G, Waks T, Eshhar Z (1989). Expression of immunoglobulin-T-cell receptor chimeric molecules as functional receptors with antibody-type specificity. Proc Natl Acad Sci U S A.

[5] Guedan S, Ruella M, June CH (2019). Emerging Cellular Therapies for Cancer. Annu Rev Immunol.

[6] Mylvaganam G, Yanez AG, Maus M, Walker BD (2019). Toward T Cell-Mediated Control or Elimination of HIV Reservoirs: Lessons From Cancer Immunology. Front Immunol.

[7] Wagner TA (2018). Quarter Century of Anti-HIV CAR T Cells. Curr HIV/AIDS Rep.

[8] Kalos M, Levine BL, Porter DL, Katz S, Grupp SA, Bagg A, June CH (2011). T cells with chimeric antigen receptors have potent antitumor effects and can establish memory in patients with advanced leukemia. Sci Transl Med.

[9] Porter DL, Levine BL, Kalos M, Bagg A, June CH (2011). Chimeric antigen receptor-modified T cells in chronic lymphoid leukemia. N Engl J Med.

[10] Liu E, Marin D, Banerjee P, Macapinlac HA, Thompson P, Basar R, Nassif Kerbauy L, Overman B, Thall P, Kaplan M (2020). Use of CAR-Transduced Natural Killer Cells in CD19-Positive Lymphoid Tumors. N Engl J Med.

[11] Garfall AL, Maus MV, Hwang WT, Lacey SF, Mahnke YD, Melenhorst JJ, Zheng Z, Vogl DT, Cohen AD, Weiss BM (2015). Chimeric Antigen Receptor T Cells against CD19 for Multiple Myeloma. N Engl J Med.

[12] Atanackovic D, Radhakrishnan SV, Bhardwaj N, Luetkens T (2016). Chimeric Antigen Receptor (CAR) therapy for multiple myeloma. Br J Haematol.

[13] Maude SL, Frey N, Shaw PA, Aplenc R, Barrett DM, Bunin NJ, Chew A, Gonzalez VE, Zheng Z, Lacey SF (2014). Chimeric antigen receptor T cells for sustained remissions in leukemia. N Engl J Med.

[14] Lee DW, Kochenderfer JN, Stetler-Stevenson M, Cui YK, Delbrook C, Feldman SA, Fry TJ, Orentas R, Sabatino M, Shah NN (2015). T cells expressing CD19 chimeric antigen receptors for acute lymphoblastic leukaemia in children and young adults: a phase 1 dose-escalation trial. Lancet.

[15] Davila ML, Riviere I, Wang X, Bartido S, Park J, Curran K, Chung SS, Stefanski J, Borquez-Ojeda O, Olszewska M (2014). Efficacy and toxicity management of 19-28z CAR T cell therapy in B cell acute lymphoblastic leukemia. Sci Transl Med.

[16] Ahmed N, Brawley VS, Hegde M, Robertson C, Ghazi A, Gerken C, Liu E, Dakhova O, Ashoori A, Corder A (2015). Human Epidermal Growth Factor Receptor 2 (HER2) -Specific Chimeric Antigen Receptor-Modified T Cells for the Immunotherapy of HER2-Positive Sarcoma. J Clin Oncol.

[17] Pule MA, Savoldo B, Myers GD, Rossig C, Russell HV, Dotti G, Huls MH, Liu E, Gee AP, Mei Z (2008). Virus-specific T cells engineered to coexpress tumor-specific receptors: persistence and antitumor activity in individuals with neuroblastoma. Nat Med.

[18] Louis CU, Savoldo B, Dotti G, Pule M, Yvon E, Myers GD, Rossig C, Russell HV, Diouf O, Liu E (2011). Antitumor activity and long-term fate of chimeric antigen receptor-positive T cells in patients with neuroblastoma. Blood.

[19] Casucci M, Hawkins RE, Dotti G, Bondanza A (2015). Overcoming the toxicity hurdles of genetically targeted T cells. Cancer Immunol Immunother.

[20] Gottschalk S, Bollard CM, Straathof KC, Louis CU, Savoldo B, Dotti G, Brenner MK, Heslop HE, Rooney CM. T cell therapies. Ernst Schering Found Symp Proc. 2006;(4):69–82. 10.1007/2789_2007_039. 10.1007/2789_2007_039 17824182

[21] Ramos CA, Savoldo B, Dotti G (2014). CD19-CAR trials. Cancer J.

[22] Savoldo B, Dotti G (2013). Chimeric antigen receptors (CARs) from bench-to-bedside. Immunol Lett.

[23] Allegra A, Innao V, Gerace D, Vaddinelli D, Musolino C (2016). Adoptive immunotherapy for hematological malignancies: Current status and new insights in chimeric antigen receptor T cells. Blood Cells Mol Dis.

[24] Liu D, Zhao J, Song Y (2019). Engineering switchable and programmable universal CARs for CAR T therapy. J Hematol Oncol.

[25] Hyrenius-Wittsten A, Roybal KT (2019). Paving New Roads for CARs. Trends Cancer.

[26] Abreu TR, Fonseca NA, Goncalves N, Moreira JN (2020). Current challenges and emerging opportunities of CAR-T cell therapies. J Control Release.

[27] Daniyan AF, Brentjens RJ. CARs of the future. Am J Hematol. 2019;94(S1):S55–8. 10.1002/ajh.25416. Epub 2019 Feb 25. 10.1002/ajh.25416 PMC8287577 30680777

[28] Shah NN, Fry TJ (2019). Mechanisms of resistance to CAR T cell therapy. Nat Rev Clin Oncol.

[29] Boyiadzis MM, Dhodapkar MV, Brentjens RJ, Kochenderfer JN, Neelapu SS, Maus MV, Porter DL, Maloney DG, Grupp SA, Mackall CL (2018). Chimeric antigen receptor (CAR) T therapies for the treatment of hematologic malignancies: clinical perspective and significance. J Immunother Cancer.

[30] Watanabe K, Kuramitsu S, Posey AD, June CH (2018). Expanding the Therapeutic Window for CAR T Cell Therapy in Solid Tumors: The Knowns and Unknowns of CAR T Cell Biology. Front Immunol.

[31] Fesnak AD, June CH, Levine BL (2016). Engineered T cells: the promise and challenges of cancer immunotherapy. Nat Rev Cancer.

[32] Ying Z, Huang XF, Xiang X, Liu Y, Kang X, Song Y, Guo X, Liu H, Ding N, Zhang T (2019). A safe and potent anti-CD19 CAR T cell therapy. Nat Med.

[33] Neelapu SS, Locke FL, Bartlett NL, Lekakis LJ, Miklos DB, Jacobson CA, Braunschweig I, Oluwole OO, Siddiqi T, Lin Y (2017). Axicabtagene Ciloleucel CAR T-Cell Therapy in Refractory Large B-Cell Lymphoma. N Engl J Med.

[34] Ramos CA, Ballard B, Zhang H, Dakhova O, Gee AP, Mei Z, Bilgi M, Wu MF, Liu H, Grilley B (2017). Clinical and immunological responses after CD30-specific chimeric antigen receptor-redirected lymphocytes. J Clin Invest.

[35] Ramos CA, Savoldo B, Torrano V, Ballard B, Zhang H, Dakhova O, Liu E, Carrum G, Kamble RT, Gee AP (2016). Clinical responses with T lymphocytes targeting malignancy-associated kappa light chains. J Clin Invest.

[36] Bonifant CL, Jackson HJ, Brentjens RJ, Curran KJ (2016). Toxicity and management in CAR T-cell therapy. Mol Ther Oncolytics.

[37] Alonso-Camino V, Harwood SL, Alvarez-Mendez A, Alvarez-Vallina L (2016). Efficacy and toxicity management of CAR-T-cell immunotherapy: a matter of responsiveness control or tumour-specificity?. Biochem Soc Trans.

[38] Kalaitsidou M, Kueberuwa G, Schutt A, Gilham DE (2015). CAR T-cell therapy: toxicity and the relevance of preclinical models. Immunotherapy.

[39] Gust J, Hay KA, Hanafi LA, Li D, Myerson D, Gonzalez-Cuyar LF, Yeung C, Liles WC, Wurfel M, Lopez JA (2017). Endothelial Activation and Blood-Brain Barrier Disruption in Neurotoxicity after Adoptive Immunotherapy with CD19 CAR-T Cells. Cancer Discov.

[40] Hay KA, Hanafi LA, Li D, Gust J, Liles WC, Wurfel MM, Lopez JA, Chen J, Chung D, Harju-Baker S (2017). Kinetics and biomarkers of severe cytokine release syndrome after CD19 chimeric antigen receptor-modified T-cell therapy. Blood.

[41] Prasad V (2018). Immunotherapy: Tisagenlecleucel - the first approved CAR-T-cell therapy: implications for payers and policy makers. Nat Rev Clin Oncol.

[42] Fooksman DR, Vardhana S, Vasiliver-Shamis G, Liese J, Blair DA, Waite J, Sacristan C, Victora GD, Zanin-Zhorov A, Dustin ML (2010). Functional anatomy of T cell activation and synapse formation. Annu Rev Immunol.

[43] Monks CR, Freiberg BA, Kupfer H, Sciaky N, Kupfer A (1998). Three-dimensional segregation of supramolecular activation clusters in T cells. Nature.

[44] Grakoui A, Bromley SK, Sumen C, Davis MM, Shaw AS, Allen PM, Dustin ML (1999). The immunological synapse: a molecular machine controlling T cell activation. Science.

[45] Campi G, Varma R, Dustin ML (2005). Actin and agonist MHC-peptide complex-dependent T cell receptor microclusters as scaffolds for signaling. J Exp Med.

[46] Varma R, Campi G, Yokosuka T, Saito T, Dustin ML (2006). T cell receptor-proximal signals are sustained in peripheral microclusters and terminated in the central supramolecular activation cluster. Immunity.

[47] Xiong W, Chen Y, Kang X, Chen Z, Zheng P, Hsu YH, Jang JH, Qin L, Liu H, Dotti G, Liu D (2018). Immunological Synapse Predicts Effectiveness of Chimeric Antigen Receptor Cells. Mol Ther.

[48] Kochenderfer JN, Somerville RPT, Lu T, Shi V, Bot A, Rossi J, Xue A, Goff SL, Yang JC, Sherry RM (2017). Lymphoma Remissions Caused by Anti-CD19 Chimeric Antigen Receptor T Cells Are Associated With High Serum Interleukin-15 Levels. J Clin Oncol.

[49] Fraietta JA, Lacey SF, Orlando EJ, Pruteanu-Malinici I, Gohil M, Lundh S, Boesteanu AC, Wang Y, O'Connor RS, Hwang WT (2018). Determinants of response and resistance to CD19 chimeric antigen receptor (CAR) T cell therapy of chronic lymphocytic leukemia. Nat Med.

[50] Vera J, Savoldo B, Vigouroux S, Biagi E, Pule M, Rossig C, Wu J, Heslop HE, Rooney CM, Brenner MK, Dotti G (2006). T lymphocytes redirected against the kappa light chain of human immunoglobulin efficiently kill mature B lymphocyte-derived malignant cells. Blood.

[51] Zhong XS, Matsushita M, Plotkin J, Riviere I, Sadelain M (2010). Chimeric antigen receptors combining 4-1BB and CD28 signaling domains augment PI3kinase/AKT/Bcl-XL activation and CD8+ T cell-mediated tumor eradication. Mol Ther.

[52] Terakura S, Yamamoto TN, Gardner RA, Turtle CJ, Jensen MC, Riddell SR (2012). Generation of CD19-chimeric antigen receptor modified CD8+ T cells derived from virus-specific central memory T cells. Blood.

[53] Dotti G, Savoldo B, Takahashi S, Goltsova T, Brown M, Rill D, Rooney C, Brenner M (2001). Adenovector-induced expression of human-CD40-ligand (hCD40L) by multiple myeloma cells. A model for immunotherapy. Exp Hematol.

[54] Jena B, Dotti G, Cooper LJ (2010). Redirecting T-cell specificity by introducing a tumor-specific chimeric antigen receptor. Blood.

[55] Hoyos V, Savoldo B, Quintarelli C, Mahendravada A, Zhang M, Vera J, Heslop HE, Rooney CM, Brenner MK, Dotti G (2010). Engineering CD19-specific T lymphocytes with interleukin-15 and a suicide gene to enhance their anti-lymphoma/leukemia effects and safety. Leukemia.

[56] Salter AI, Ivey RG, Kennedy JJ, Voillet V, Rajan A, Alderman EJ, Voytovich UJ, Lin C, Sommermeyer D, Liu L, et al. Phosphoproteomic analysis of chimeric antigen receptor signaling reveals kinetic and quantitative differences that affect cell function. Sci Signal. 2018;11(544):eaat6753. 10.1126/scisignal.aat6753. 10.1126/scisignal.aat6753 PMC6186424 30131370

[57] Morse D, Tannous BA (2012). A water-soluble coelenterazine for sensitive in vivo imaging of coelenterate luciferases. Mol Ther.

[58] Wang H, Cao F, De A, Cao Y, Contag C, Gambhir SS, Wu JC, Chen X (2009). Trafficking mesenchymal stem cell engraftment and differentiation in tumor-bearing mice by bioluminescence imaging. Stem Cells.

[59] Bhaumik S, Gambhir SS (2002). Optical imaging of Renilla luciferase reporter gene expression in living mice. Proc Natl Acad Sci U S A.

[60] Kim YJ, Dubey P, Ray P, Gambhir SS, Witte ON (2004). Multimodality imaging of lymphocytic migration using lentiviral-based transduction of a tri-fusion reporter gene. Mol Imaging Biol.

[61] Srivastava S, Riddell SR (2018). Chimeric Antigen Receptor T Cell Therapy: Challenges to Bench-to-Bedside Efficacy. J Immunol.

[62] Johnson LA, June CH (2017). Driving gene-engineered T cell immunotherapy of cancer. Cell Res.

[63] Liu D, Tian S, Zhang K, Xiong W, Lubaki NM, Chen Z, Han W (2017). Chimeric antigen receptor (CAR)-modified natural killer cell-based immunotherapy and immunological synapse formation in cancer and HIV. Protein Cell.

[64] Jang JH, Huang Y, Zheng P, Jo MC, Bertolet G, Zhu MX, Qin L, Liu D (2015). Imaging of Cell-Cell Communication in a Vertical Orientation Reveals High-Resolution Structure of Immunological Synapse and Novel PD-1 Dynamics. J Immunol.

[65] Singh N, Liu X, Hulitt J, Jiang S, June CH, Grupp SA, Barrett DM, Zhao Y (2014). Nature of tumor control by permanently and transiently modified GD2 chimeric antigen receptor T cells in xenograft models of neuroblastoma. Cancer Immunol Res.

[66] Yvon E, Del Vecchio M, Savoldo B, Hoyos V, Dutour A, Anichini A, Dotti G, Brenner MK (2009). Immunotherapy of metastatic melanoma using genetically engineered GD2-specific T cells. Clin Cancer Res.

[67] Zheng P, Bertolet G, Chen Y, Huang S, Liu D. Super-resolution imaging of the natural killer cell immunological synapse on a glass-supported planar lipid bilayer. J Vis Exp. 2015;(96):52502. 10.3791/52502. 10.3791/52502 PMC4354632 25741636

[68] Fraietta JA, Nobles CL, Sammons MA, Lundh S, Carty SA, Reich TJ, Cogdill AP, Morrissette JJD, DeNizio JE, Reddy S (2018). Disruption of TET2 promotes the therapeutic efficacy of CD19-targeted T cells. Nature.

[69] Nobles CL, Sherrill-Mix S, Everett JK, Reddy S, Fraietta JA, Porter DL, Frey N, Gill SI, Grupp SA, Maude SL (2020). CD19-targeting CAR T cell immunotherapy outcomes correlate with genomic modification by vector integration. J Clin Invest.

[70] Lim WA, June CH (2017). The Principles of Engineering Immune Cells to Treat Cancer. Cell.

[71] Depil S, Duchateau P, Grupp SA, Mufti G. Poirot L: ‘Off-the-shelf’ allogeneic CAR T cells: development and challenges. Nat Rev Drug Discov. 2020;19(3):185–99. 10.1038/s41573-019-0051-2. Epub 2020 Jan 3. 10.1038/s41573-019-0051-2 31900462

[72] Baragano Raneros A, Lopez-Larrea C, Suarez-Alvarez B (2019). Acute myeloid leukemia and NK cells: two warriors confront each other. Oncoimmunology.

[73] Hawkes N (2016). Trial of novel leukaemia drug is stopped for second time after two more deaths. BMJ.

[74] Abbasi J (2017). Amid FDA Approval Filings, Another CAR-T Therapy Patient Death. JAMA.

[75] Brudno JN, Kochenderfer JN (2016). Toxicities of chimeric antigen receptor T cells: recognition and management. Blood.

[76] Sun S, Hao H, Yang G, Zhang Y, Fu Y (2018). Immunotherapy with CAR-Modified T Cells: Toxicities and Overcoming Strategies. J Immunol Res.

[77] Brentjens RJ, Davila ML, Riviere I, Park J, Wang X, Cowell LG, Bartido S, Stefanski J, Taylor C, Olszewska M (2013). CD19-targeted T cells rapidly induce molecular remissions in adults with chemotherapy-refractory acute lymphoblastic leukemia. Sci Transl Med.

[78] Shimabukuro-Vornhagen A, Godel P, Subklewe M, Stemmler HJ, Schlosser HA, Schlaak M, Kochanek M, Boll B, von Bergwelt-Baildon MS (2018). Cytokine release syndrome. J Immunother Cancer.

[79] Godel P, Shimabukuro-Vornhagen A, von Bergwelt-Baildon M (2018). Understanding cytokine release syndrome. Intensive Care Med.

[80] Barrett DM, Teachey DT, Grupp SA (2014). Toxicity management for patients receiving novel T-cell engaging therapies. Curr Opin Pediatr.

[81] Norelli M, Camisa B, Barbiera G, Falcone L, Purevdorj A, Genua M, Sanvito F, Ponzoni M, Doglioni C, Cristofori P (2018). Monocyte-derived IL-1 and IL-6 are differentially required for cytokine-release syndrome and neurotoxicity due to CAR T cells. Nat Med.

[82] Giavridis T, van der Stegen SJC, Eyquem J, Hamieh M, Piersigilli A, Sadelain M (2018). CAR T cell-induced cytokine release syndrome is mediated by macrophages and abated by IL-1 blockade. Nat Med.

[83] Staedtke V, Bai RY, Kim K, Darvas M, Davila ML, Riggins GJ, Rothman PB, Papadopoulos N, Kinzler KW, Vogelstein B, Zhou S (2018). Disruption of a self-amplifying catecholamine loop reduces cytokine release syndrome. Nature.

[84] Baymon DE, Boyer EW (2019). Chimeric antigen receptor T-cell toxicity. Curr Opin Pediatr.

[85] Turtle CJ, Hanafi LA, Berger C, Gooley TA, Cherian S, Hudecek M, Sommermeyer D, Melville K, Pender B, Budiarto TM (2016). CD19 CAR-T cells of defined CD4+:CD8+ composition in adult B cell ALL patients. J Clin Invest.

[86] Porter DL, Hwang WT, Frey NV, Lacey SF, Shaw PA, Loren AW, Bagg A, Marcucci KT, Shen A, Gonzalez V (2015). Chimeric antigen receptor T cells persist and induce sustained remissions in relapsed refractory chronic lymphocytic leukemia. Sci Transl Med.

[87] Brudno JN, Somerville RP, Shi V, Rose JJ, Halverson DC, Fowler DH, Gea-Banacloche JC, Pavletic SZ, Hickstein DD, Lu TL (2016). Allogeneic T Cells That Express an Anti-CD19 Chimeric Antigen Receptor Induce Remissions of B-Cell Malignancies That Progress After Allogeneic Hematopoietic Stem-Cell Transplantation Without Causing Graft-Versus-Host Disease. J Clin Oncol.

[88] Kochenderfer JN, Dudley ME, Kassim SH, Somerville RP, Carpenter RO, Stetler-Stevenson M, Yang JC, Phan GQ, Hughes MS, Sherry RM (2015). Chemotherapy-refractory diffuse large B-cell lymphoma and indolent B-cell malignancies can be effectively treated with autologous T cells expressing an anti-CD19 chimeric antigen receptor. J Clin Oncol.

[89] Neelapu SS, Tummala S, Kebriaei P, Wierda W, Gutierrez C, Locke FL, Komanduri KV, Lin Y, Jain N, Daver N (2018). Chimeric antigen receptor T-cell therapy - assessment and management of toxicities. Nat Rev Clin Oncol.

[90] Grupp SA, Kalos M, Barrett D, Aplenc R, Porter DL, Rheingold SR, Teachey DT, Chew A, Hauck B, Wright JF (2013). Chimeric antigen receptor-modified T cells for acute lymphoid leukemia. N Engl J Med.

[91] Mei H, Jiang H, Wu Y, Guo T, Xia L, Jin R, Hu Y (2018). Neurological toxicities and coagulation disorders in the cytokine release syndrome during CAR-T therapy. Br J Haematol.

[92] Pennell CA, Barnum JL, McDonald-Hyman CS, Panoskaltsis-Mortari A, Riddle MJ, Xiong Z, Loschi M, Thangavelu G, Campbell HM, Storlie MD (2018). Human CD19-Targeted Mouse T Cells Induce B Cell Aplasia and Toxicity in Human CD19 Transgenic Mice. Mol Ther.

[93] Ruella M, June CH (2018). Predicting Dangerous Rides in CAR T Cells: Bridging the Gap between Mice and Humans. Mol Ther.

[94] Lee DW, Gardner R, Porter DL, Louis CU, Ahmed N, Jensen M, Grupp SA, Mackall CL (2014). Current concepts in the diagnosis and management of cytokine release syndrome. Blood.

[95] Maude SL, Barrett D, Teachey DT, Grupp SA (2014). Managing cytokine release syndrome associated with novel T cell-engaging therapies. Cancer J.

[96] Kochenderfer JN, Dudley ME, Carpenter RO, Kassim SH, Rose JJ, Telford WG, Hakim FT, Halverson DC, Fowler DH, Hardy NM (2013). Donor-derived CD19-targeted T cells cause regression of malignancy persisting after allogeneic hematopoietic stem cell transplantation. Blood.

[97] McKane M, Soslow JH, Xu M, Saville BR, Slaughter JC, Burnette WB, Markham LW (2017). Does Body Mass Index Predict Premature Cardiomyopathy Onset for Duchenne Muscular Dystrophy?. J Child Neurol.

[98] Segura LG, Lorenz JD, Weingarten TN, Scavonetto F, Bojanic K, Selcen D, Sprung J (2013). Anesthesia and Duchenne or Becker muscular dystrophy: review of 117 anesthetic exposures. Paediatr Anaesth.

[99] Kochenderfer JN, Dudley ME, Feldman SA, Wilson WH, Spaner DE, Maric I, Stetler-Stevenson M, Phan GQ, Hughes MS, Sherry RM (2012). B-cell depletion and remissions of malignancy along with cytokine-associated toxicity in a clinical trial of anti-CD19 chimeric-antigen-receptor-transduced T cells. Blood.

[100] Kochenderfer JN, Wilson WH, Janik JE, Dudley ME, Stetler-Stevenson M, Feldman SA, Maric I, Raffeld M, Nathan DA, Lanier BJ (2010). Eradication of B-lineage cells and regression of lymphoma in a patient treated with autologous T cells genetically engineered to recognize CD19. Blood.

[101] Timmers M, Roex G, Wang Y, Campillo-Davo D, Van Tendeloo VFI, Chu Y, Berneman ZN, Luo F, Van Acker HH, Anguille S (2019). Chimeric Antigen Receptor-Modified T Cell Therapy in Multiple Myeloma: Beyond B Cell Maturation Antigen. Front Immunol.

[102] Bukhari A, El Chaer F, Koka R, Singh Z, Hutnick E, Ruehle K, Lee ST, Kocoglu MH, Shanholtz C, Badros A, et al. Rapid relapse of large B-cell lymphoma after CD19 directed CAR-T-cell therapy due to CD-19 antigen loss. Am J Hematol. 2019;94(10):E273–5. 10.1002/ajh.25591. 10.1002/ajh.25591 31342556

[103] Brown CE, Alizadeh D, Starr R, Weng L, Wagner JR, Naranjo A, Ostberg JR, Blanchard MS, Kilpatrick J, Simpson J (2016). Regression of Glioblastoma after Chimeric Antigen Receptor T-Cell Therapy. N Engl J Med.

[104] Pegram HJ, Lee JC, Hayman EG, Imperato GH, Tedder TF, Sadelain M, Brentjens RJ (2012). Tumor-targeted T cells modified to secrete IL-12 eradicate systemic tumors without need for prior conditioning. Blood.

[105] Cruz CR, Micklethwaite KP, Savoldo B, Ramos CA, Lam S, Ku S, Diouf O, Liu E, Barrett AJ, Ito S (2013). Infusion of donor-derived CD19-redirected virus-specific T cells for B-cell malignancies relapsed after allogeneic stem cell transplant: a phase 1 study. Blood.

[106] Fry TJ, Shah NN, Orentas RJ, Stetler-Stevenson M, Yuan CM, Ramakrishna S, Wolters P, Martin S, Delbrook C, Yates B (2018). CD22-targeted CAR T cells induce remission in B-ALL that is naive or resistant to CD19-targeted CAR immunotherapy. Nat Med.

[107] Orlando EJ, Han X, Tribouley C, Wood PA, Leary RJ, Riester M, Levine JE, Qayed M, Grupp SA, Boyer M (2018). Genetic mechanisms of target antigen loss in CAR19 therapy of acute lymphoblastic leukemia. Nat Med.

[108] Xu X, Sun Q, Liang X, Chen Z, Zhang X, Zhou X, Li M, Tu H, Liu Y, Tu S, Li Y (2019). Mechanisms of Relapse After CD19 CAR T-Cell Therapy for Acute Lymphoblastic Leukemia and Its Prevention and Treatment Strategies. Front Immunol.

[109] Li J, Li W, Huang K, Zhang Y, Kupfer G, Zhao Q (2018). Chimeric antigen receptor T cell (CAR-T) immunotherapy for solid tumors: lessons learned and strategies for moving forward. J Hematol Oncol.

[110] van der Stegen SJ, Hamieh M, Sadelain M (2015). The pharmacology of second-generation chimeric antigen receptors. Nat Rev Drug Discov.

[111] Sadelain M, Riviere I, Riddell S (2017). Therapeutic T cell engineering. Nature.

[112] Jaspers JE, Brentjens RJ (2017). Development of CAR T cells designed to improve antitumor efficacy and safety. Pharmacol Ther.

[113] Morgan RA, Yang JC, Kitano M, Dudley ME, Laurencot CM, Rosenberg SA (2010). Case report of a serious adverse event following the administration of T cells transduced with a chimeric antigen receptor recognizing ERBB2. Mol Ther.

[114] Newick K, O'Brien S, Moon E, Albelda SM (2017). CAR T Cell Therapy for Solid Tumors. Annu Rev Med.

[115] Yu S, Li A, Liu Q, Li T, Yuan X, Han X, Wu K (2017). Chimeric antigen receptor T cells: a novel therapy for solid tumors. J Hematol Oncol.

[116] Schmidts A, Maus MV (2018). Making CAR T Cells a Solid Option for Solid Tumors. Front Immunol.

[117] Tahmasebi S, Elahi R, Esmaeilzadeh A (2019). Solid Tumors Challenges and New Insights of CAR T Cell Engineering. Stem Cell Rev Rep.

[118] Morgan MA, Schambach A (2018). Engineering CAR-T Cells for Improved Function Against Solid Tumors. Front Immunol.

[119] Poznansky MC, Olszak IT, Foxall R, Evans RH, Luster AD, Scadden DT (2000). Active movement of T cells away from a chemokine. Nat Med.

[120] Feig C, Jones JO, Kraman M, Wells RJ, Deonarine A, Chan DS, Connell CM, Roberts EW, Zhao Q, Caballero OL (2013). Targeting CXCL12 from FAP-expressing carcinoma-associated fibroblasts synergizes with anti-PD-L1 immunotherapy in pancreatic cancer. Proc Natl Acad Sci U S A.

[121] Buckanovich RJ, Facciabene A, Kim S, Benencia F, Sasaroli D, Balint K, Katsaros D, O'Brien-Jenkins A, Gimotty PA, Coukos G (2008). Endothelin B receptor mediates the endothelial barrier to T cell homing to tumors and disables immune therapy. Nat Med.

[122] Craddock JA, Lu A, Bear A, Pule M, Brenner MK, Rooney CM, Foster AE (2010). Enhanced tumor trafficking of GD2 chimeric antigen receptor T cells by expression of the chemokine receptor CCR2b. J Immunother.

[123] Newick K, O'Brien S, Sun J, Kapoor V, Maceyko S, Lo A, Pure E, Moon E, Albelda SM (2016). Augmentation of CAR T-cell Trafficking and Antitumor Efficacy by Blocking Protein Kinase A Localization. Cancer Immunol Res.

[124] Salmon H, Franciszkiewicz K, Damotte D, Dieu-Nosjean MC, Validire P, Trautmann A, Mami-Chouaib F, Donnadieu E (2012). Matrix architecture defines the preferential localization and migration of T cells into the stroma of human lung tumors. J Clin Invest.

[125] Rabinovich GA, Gabrilovich D, Sotomayor EM (2007). Immunosuppressive strategies that are mediated by tumor cells. Annu Rev Immunol.

[126] Martinez M, Moon EK (2019). CAR T Cells for Solid Tumors: New Strategies for Finding, Infiltrating, and Surviving in the Tumor Microenvironment. Front Immunol.

[127] Chong EA, Melenhorst JJ, Lacey SF, Ambrose DE, Gonzalez V, Levine BL, June CH, Schuster SJ (2017). PD-1 blockade modulates chimeric antigen receptor (CAR)-modified T cells: refueling the CAR. Blood.

[128] Heczey A, Louis CU, Savoldo B, Dakhova O, Durett A, Grilley B, Liu H, Wu MF, Mei Z, Gee A (2017). CAR T Cells Administered in Combination with Lymphodepletion and PD-1 Inhibition to Patients with Neuroblastoma. Mol Ther.

[129] Hu W, Zi Z, Jin Y, Li G, Shao K, Cai Q, Ma X, Wei F (2019). CRISPR/Cas9-mediated PD-1 disruption enhances human mesothelin-targeted CAR T cell effector functions. Cancer Immunol Immunother.

[130] Yoon DH, Osborn MJ, Tolar J, Kim CJ. Incorporation of Immune Checkpoint Blockade into Chimeric Antigen Receptor T Cells (CAR-Ts): Combination or Built-In CAR-T. Int J Mol Sci. 2018;19(2):340. 10.3390/ijms19020340. 10.3390/ijms19020340 PMC5855562 29364163

[131] Condomines M, Arnason J, Benjamin R, Gunset G, Plotkin J, Sadelain M (2015). Tumor-Targeted Human T Cells Expressing CD28-Based Chimeric Antigen Receptors Circumvent CTLA-4 Inhibition. PLoS One.

[132] Dong H, Strome SE, Salomao DR, Tamura H, Hirano F, Flies DB, Roche PC, Lu J, Zhu G, Tamada K (2002). Tumor-associated B7-H1 promotes T-cell apoptosis: a potential mechanism of immune evasion. Nat Med.

[133] Muliaditan T, Opzoomer JW, Caron J, Okesola M, Kosti P, Lall S, Van Hemelrijck M, Dazzi F, Tutt A, Grigoriadis A (2018). Repurposing Tin Mesoporphyrin as an Immune Checkpoint Inhibitor Shows Therapeutic Efficacy in Preclinical Models of Cancer. Clin Cancer Res.

[134] Sharma P, Allison JP (2015). Immune checkpoint targeting in cancer therapy: toward combination strategies with curative potential. Cell.

[135] Rafiq S, Yeku OO, Jackson HJ, Purdon TJ, van Leeuwen DG, Drakes DJ, Song M, Miele MM, Li Z, Wang P (2018). Targeted delivery of a PD-1-blocking scFv by CAR-T cells enhances anti-tumor efficacy in vivo. Nat Biotechnol.

[136] Liu X, Ranganathan R, Jiang S, Fang C, Sun J, Kim S, Newick K, Lo A, June CH, Zhao Y, Moon EK (2016). A Chimeric Switch-Receptor Targeting PD1 Augments the Efficacy of Second-Generation CAR T Cells in Advanced Solid Tumors. Cancer Res.

[137] Wei J, Luo C, Wang Y, Guo Y, Dai H, Tong C, Ti D, Wu Z, Han W (2019). PD-1 silencing impairs the anti-tumor function of chimeric antigen receptor modified T cells by inhibiting proliferation activity. J Immunother Cancer.

[138] Chmielewski M, Abken H (2017). CAR T Cells Releasing IL-18 Convert to T-Bet (high) FoxO1(low) Effectors that Exhibit Augmented Activity against Advanced Solid Tumors. Cell Rep.

[139] Yeku OO, Brentjens RJ (2016). Armored CAR T-cells: utilizing cytokines and pro-inflammatory ligands to enhance CAR T-cell anti-tumour efficacy. Biochem Soc Trans.

[140] Seo N, Hayakawa S, Takigawa M, Tokura Y (2001). Interleukin-10 expressed at early tumour sites induces subsequent generation of CD4(+) T-regulatory cells and systemic collapse of antitumour immunity. Immunology.

[141] Jarnicki AG, Lysaght J, Todryk S, Mills KH (2006). Suppression of antitumor immunity by IL-10 and TGF-beta-producing T cells infiltrating the growing tumor: influence of tumor environment on the induction of CD4+ and CD8+ regulatory T cells. J Immunol.

[142] Chu F, Cao J, Neelalpu SS (2018). Versatile CAR T-cells for cancer immunotherapy. Contemp Oncol (Pozn).

[143] Xu X, Gnanaprakasam JNR, Sherman J, Wang R (2019). A Metabolism Toolbox for CAR T Therapy. Front Oncol.

[144] DeBerardinis RJ, Chandel NS (2016). Fundamentals of cancer metabolism. Sci Adv.

[145] Kosti P, Maher J, Arnold JN (2018). Perspectives on Chimeric Antigen Receptor T-Cell Immunotherapy for Solid Tumors. Front Immunol.

[146] Ladika S (2019). Beware of the CAR-T Hitches. Manag Care.

[147] Zhao J, Lin Q, Song Y, Liu D (2018). Universal CARs, universal T cells, and universal CAR T cells. J Hematol Oncol.

[148] Zabel M, Tauber PA, Pickl WF (2019). The making and function of CAR cells. Immunol Lett.

[149] Saetersmoen ML, Hammer Q, Valamehr B, Kaufman DS, Malmberg KJ (2019). Off-the-shelf cell therapy with induced pluripotent stem cell-derived natural killer cells. Semin Immunopathol.

[150] Liu E, Tong Y, Dotti G, Shaim H, Savoldo B, Mukherjee M, Orange J, Wan X, Lu X, Reynolds A (2018). Cord blood NK cells engineered to express IL-15 and a CD19-targeted CAR show long-term persistence and potent antitumor activity. Leukemia.

[151] Kriegsmann K, Kriegsmann M, von Bergwelt-Baildon M, Cremer M, Witzens-Harig M (2018). NKT cells - New players in CAR cell immunotherapy?. Eur J Haematol.

[152] Keir ME, Butte MJ, Freeman GJ, Sharpe AH (2008). PD-1 and its ligands in tolerance and immunity. Annu Rev Immunol.

[153] John LB, Kershaw MH, Darcy PK (2013). Blockade of PD-1 immunosuppression boosts CAR T-cell therapy. Oncoimmunology.

[154] Cherkassky L, Morello A, Villena-Vargas J, Feng Y, Dimitrov DS, Jones DR, Sadelain M, Adusumilli PS (2016). Human CAR T cells with cell-intrinsic PD-1 checkpoint blockade resist tumor-mediated inhibition. J Clin Invest.

[155] Chong EA, Melenhorst JJ, Lacey SF, Ambrose DE, Gonzalez V, Levine B, June CH, Schuster SJ. PD-1 Blockade Modulates Chimeric Antigen Receptor (CAR) Modified T Cells and Induces Tumor Regression: Refueling the CAR. Blood. 2017;129(8):1039–41. 10.1182/blood-2016-09-738245. 10.1182/blood-2016-09-738245 PMC5391777 28031179

[156] Gargett T, Yu W, Dotti G, Yvon ES, Christo SN, Hayball JD, Lewis ID, Brenner MK, Brown MP (2016). GD2-specific CAR T Cells Undergo Potent Activation and Deletion Following Antigen Encounter but can be Protected From Activation-induced Cell Death by PD-1 Blockade. Mol Ther.

